# An SPNS1-dependent lysosomal lipid transport pathway that enables cell survival under choline limitation

**DOI:** 10.1126/sciadv.adf8966

**Published:** 2023-04-19

**Authors:** Samantha G. Scharenberg, Wentao Dong, Ali Ghoochani, Kwamina Nyame, Roni Levin-Konigsberg, Aswini R. Krishnan, Eshaan S. Rawat, Kaitlyn Spees, Michael C. Bassik, Monther Abu-Remaileh

**Affiliations:** ^1^ Department of Chemical Engineering, Stanford University, Stanford, CA 94305, USA.; ^2^ Department of Genetics, Stanford University, Stanford, CA 94305, USA.; ^3^ The Institute for Chemistry, Engineering and Medicine for Human Health (Sarafan ChEM-H), Stanford University, Stanford, CA 94305, USA.; ^4^ Stanford Medical Scientist Training Program, Stanford University, Stanford, CA 94305, USA.; ^5^ Stanford Biophysics Program, Stanford University, Stanford, CA 94305, USA.; ^6^ Department of Biochemistry, Stanford University School of Medicine, Stanford, CA 94305, USA.

## Abstract

Lysosomes degrade macromolecules and recycle their nutrient content to support cell function and survival. However, the machineries involved in lysosomal recycling of many nutrients remain to be discovered, with a notable example being choline, an essential metabolite liberated via lipid degradation. Here, we engineered metabolic dependency on lysosome-derived choline in pancreatic cancer cells to perform an endolysosome-focused CRISPR-Cas9 screen for genes mediating lysosomal choline recycling. We identified the orphan lysosomal transmembrane protein SPNS1 as critical for cell survival under choline limitation. SPNS1 loss leads to intralysosomal accumulation of lysophosphatidylcholine (LPC) and lysophosphatidylethanolamine (LPE). Mechanistically, we reveal that SPNS1 is a proton gradient–dependent transporter of LPC species from the lysosome for their re-esterification into phosphatidylcholine in the cytosol. Last, we establish that LPC efflux by SPNS1 is required for cell survival under choline limitation. Collectively, our work defines a lysosomal phospholipid salvage pathway that is essential under nutrient limitation and, more broadly, provides a robust platform to deorphan lysosomal gene function.

## Teaser

A lysosomal pathway for lipid salvage that relies on SPNS1-mediated lysophospholipid transport is vital during nutrient scarcity.

## INTRODUCTION

Cells require a constant supply of nutrients to support their vital activities and growth. To survive inevitable fluctuations in nutrient availability, eukaryotes have evolved multiple strategies to acquire small molecules essential for their survival (*1*). Important among these are the endocytic pathways that enable internalization of nutrient-rich cargo that cannot translocate across the plasma membrane by passive diffusion or active transport. These pathways converge on the lysosome, which scavenges the vital nutrients by degrading endocytosed cargos and liberating their constituent metabolites (*2*). Thus, nutrient recycling through the lysosome represents a critical source of metabolic adaptability for cells to survive various states of nutrient deprivation.

Despite their essential role in nutrient acquisition, the lysosomal machineries that mediate nutrient recycling, in particular, the transport of liberated nutrients to the cytosol, are not fully defined. In recent work, we demonstrated that the lysosomal transporter SLC38A9 becomes selectively essential for cell growth under leucine-scarce conditions, while it is dispensable under leucine-replete conditions, leading to the discovery that SLC38A9 functions to efflux leucine derived from lysosome-degraded proteins (*3*). This work illustrates that metabolic dependency on lysosomes engineered through depleting culture medium of essential nutrients can be exploited to find lysosomal proteins involved in their recycling.

**Fig. 1.**
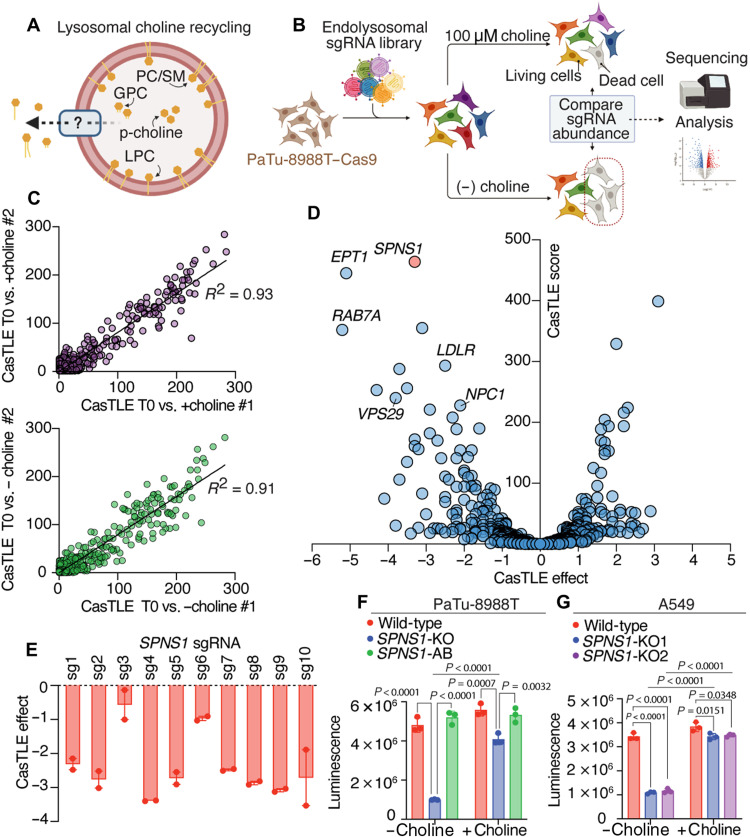
Endolysosomal CRISPR-Cas9 screen implicates *SPNS1* in the response to choline deprivation. (**A**) Overview of lysosomal choline recycling highlighting an unknown transporter that exports choline-containing catabolites. p-choline, phosphocholine. (**B**) Schematic overview of the endolysosomal CRISPR-Cas9–negative selection screen performed to identify genes required for lysosomal choline recycling. (**C**) Correlation plots between biological replicates in the endolysosomal screen for each culture condition (top, +choline condition; bottom, −choline condition). Plots depict CasTLE scores computed between time 0 and 14 doublings for each replicate under the indicated culture condition. Simple linear regression was performed with *R*^2^ values indicated on the plots. (**D**) Volcano plot for −choline versus +choline CasTLE score versus the effect for each gene in the endolysosomal library (CasTLE effect) (*n* = 2). Endolysosomal genes that are essential under the −choline condition have a negative CasTLE effect size. The highest-scoring gene (*SPNS1*) is highlighted in red. Tabulated data are found in table S2. (**E**) Individual CasTLE effects for −choline versus +choline for all *SPNS1*-targeting sgRNAs in the endolysosomal library. Data show means ± SEM (*n* = 2). (**F**) Live-cell abundance measured by CellTiter-Glo of wild-type, *SPNS1*-KO, and *SPNS1*-AB PaTu-8988T cells after 5 days in −choline and +choline cultures. Data show means ± SEM (*n* = 3). Statistical test: One-way analysis of variance (ANOVA) with Tukey’s post hoc test. (**G**) Live-cell abundance measured by CellTiter-Glo of wild-type, *SPNS1*-KO1, and *SPNS1*-KO2 A549 cells after 5 days in −choline and +choline cultures. Data show means ± SEM (*n* = 3). Statistical test: One-way ANOVA with Tukey’s post hoc test.

One essential nutrient recycled through the lysosome is choline (*4*, *5*). Choline is a small, cationic metabolite that constitutes the polar head group of the most abundant cellular phospholipid phosphatidylcholine (PC), which comprises >50% of cellular membranes, and sphingomyelin (SM). It is also required for the synthesis of the neurotransmitter acetylcholine and serves as a methyl donor in one-carbon metabolism (*6*). Through the degradation of choline-containing lipids, large quantities of choline-containing catabolites are produced and exported from the lysosome to be recycled in the cytosol (Fig. 1A) (*7*, *8*). Despite their high flux through the lysosome, many questions remain about the identity of the choline-containing compounds being recycled and the machineries mediating this process.

Here, we apply a customized CRISPR-Cas9 screen coupled with engineered choline-depleted culture medium to identify a choline-phospholipid salvage pathway orchestrated by the lysosome. We find that lysosomes enable cell survival under choline deprivation by supplying lysophospholipids, in particular, lysophosphatidylcholine (LPC), to augment phospholipid production in the cytoplasm. This mechanism is dependent on the orphan lysosomal major facilitator superfamily transporter SPNS1, the loss of which leads to substance storage and neurodegeneration-like disease in animal models (*6*, *9*–*12*). We demonstrate that SPNS1 functions to export LPC species from the lysosomal lumen to the cytosol for reacylation to PC. By supplying choline-containing scaffolds for PC synthesis through SPNS1, lysosomal LPCs allow cell survival under exogenous choline limitation. This work thus reveals a previously unknown pathway for lysophospholipid export from the lysosome that supports synthesis of vital phospholipids and, more broadly, establishes a generalizable approach for deorphanizing lysosomal genes and pathways.

## RESULTS

### A forward genetics approach reveals *SPNS1* as an essential lysosomal gene under choline-limited conditions

We speculated that growing cells in medium depleted of free choline would force their dependence on lysosomal recycling of choline, thereby conferring essentiality to lysosomal choline-scavenging machineries. This engineered dependency, coupled with high-throughput negative-selection genetic screening, might represent an efficient, unbiased approach to identifying lysosomal genes involved in recycling choline-containing molecules.

To this end, we designed a CRISPR-Cas9 library targeting all endolysosomal genes and a select subset of metabolic and signaling genes [1061 genes with 10 single-guide RNAs (sgRNAs) per gene, endolysosomal library hereafter] (see Materials and Methods and table S1). Using this library, we sought to determine endolysosomal genes involved in choline recycling by screening for those that become essential under choline deprivation (Fig. 1, A and B). We selected the pancreatic cancer cell line PaTu-8988T for screening because these cells survive choline deprivation while still exhibiting modest sensitivity to choline restriction (fig. S1, A and B), indicating an active choline scavenging pathway. This behavior is consistent with the pancreatic ductal adenocarcinoma origin of PaTu-8988T cells, a cancer known for adapting to nutrient-scarce microenvironment (*13*–*16*).

PaTu-8988T cells expressing Cas9 were infected with the endolysosomal library and propagated for 14 doublings in culture medium lacking free choline (−choline) or supplemented with 100 μM choline (+choline) (Fig. 1B). A high correlation between replicates under both the −choline and +choline conditions was observed (Fig. 1C). Gene scores and depletion/enrichment effects between the −choline and +choline conditions were determined using Cas9 high-Throughput maximum Likelihood Estimator (CasTLE) analysis (*17*), revealing genes whose targeting sgRNAs are enriched or depleted under the −choline condition (Fig. 1D and table S2). Genes whose targeting sgRNAs are depleted under the −choline condition are considered essential for cells to grow in the absence of exogenous choline. Among the highly scoring genes in this category is *EPT1*, a phosphotransferase that catalyzes the synthesis of the phospholipid phosphatidylethanolamine (PE), a direct lipid precursor of PC (*18*). PE is trimethylated to PC by the enzyme PE *N*-methyltransferase (encoded by *PEMT*) and serves as an important alternative substrate for PC synthesis in the absence of free choline (*6*); moreover, PC synthesized from PE can be catabolized to produce free choline. Thus, *EPT1* represents a positive control in this screen (Fig. 1D) (*18*–*20*).

In addition, a cluster of genes necessary for trafficking macromolecules to the lysosome were identified to be required for cell growth under the −choline condition (Fig. 1D). Of these are *RAB7A*, encoding a small guanosine triphosphatase responsible for regulating late endocytic transport to the lysosome (*21*, *22*); *LDLR*, encoding the low-density lipoprotein receptor (LDLR) that mediates the uptake and delivery of lipid-rich extracellular lipoproteins to the lysosome (*23*, *24*); and *VPS29*, encoding a retromer complex component necessary for recycling transmembrane proteins, including LDLR, from degradation pathways back to the cell surface for continuous cargo trafficking to the lysosome (*25*). Together, these results indicate that faithful trafficking of choline-containing cargo (mainly phospholipids) to lysosomes is essential for survival under choline-restricted conditions, supporting an essential role for lysosome-mediated phospholipid catabolism in compensating for choline restriction.

To identify the elusive lysosomal choline-containing catabolite transporter, we focused on transmembrane proteins. *NPC1*, encoding the lysosomal permease for cholesterol and, putatively, sphingosine (*26*–*28*) became more essential under the −choline condition (Fig. 1D and fig. S1C). *NPC1* loss causes Niemann-Pick type C, a fatal autosomal-recessive lipid-storage disorder characterized by progressive neurodegeneration (*29*). *NPC1* deficiency is known to disrupt SM catabolism (*27*), a potential source of choline, which might explain its essentiality under the −choline condition. Notably, the top-scoring gene whose targeting guides were depleted under the −choline condition was *SPNS1* (Fig. 1D), which encodes SPNS1, a lysosomal transmembrane protein and a member of the major facilitator superfamily previously hypothesized to export sugars (*9*) or sphingolipids (*30*). Deficiency in *SPNS1* homologs results in neurodegeneration and lysosomal storage disease-like presentation in model organisms (*9*–*12*), mirroring biochemical pathologies associated with deficiency of lysosomal solute carriers. All 10 sgRNAs targeting *SPNS1* were more depleted under the −choline compared to +choline condition, indicating the robustness of the phenotype resulting from SPNS1 depletion (Fig. 1E).

To validate that *SPNS1* is an essential gene during free choline restriction, we generated *SPNS1*–knockout (KO) cells in two cell lines: PaTu-8988T pancreatic cancer cells and A549 lung adenocarcinoma cells (fig. S1, D and E). We compared growth between *SPNS1-*KO cells and wild-type cells cultured under −choline and +choline conditions for 5 days. Consistent with our screen results, we observed a marked growth defect in *SPNS1*-KO cells compared to wild-type cells under –choline condition compared to only minor defect under +choline condition for both PaTu-8988T and A549 lines (Fig. 1, F and G). Reexpressing *SPNS1* in *SPNS1*-KO cells (*SPNS1*-addback, hereafter *SPNS1*-AB) completely rescued cell growth defects (Fig. 1F and fig. S1F). Together, these results indicate that SPNS1 function is required for cell growth under choline-restricted conditions.

### Loss of *SPNS1* leads to cell death under choline deprivation

**Fig. 2.**
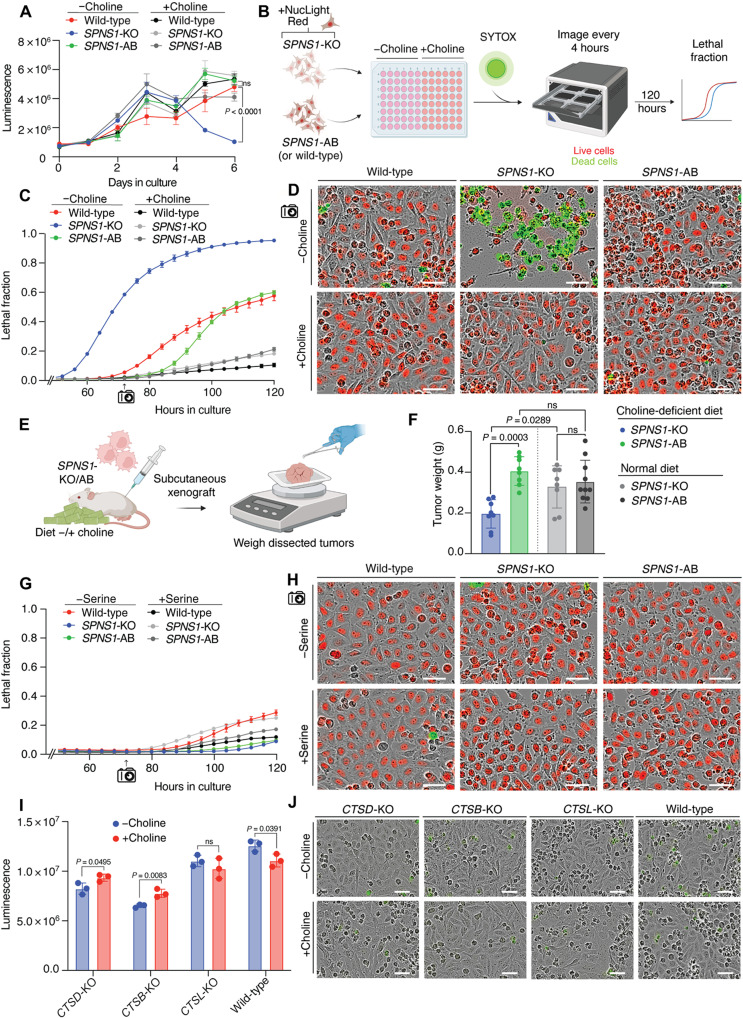
*SPNS1* deficiency promotes cell death under choline-limiting conditions. (**A**) Growth curves for wild-type, *SPNS1*-KO, and *SPNS1*-AB PaTu-8988T cells in choline-depleted medium (−choline) or medium supplemented with 100 μM choline (+choline). Data show means ± SEM (*n* = 3). Statistical test: Two-tailed unpaired *t* test. (**B**) Incucyte workflow for generating lethal fraction curves. (**C**) Lethal fraction of wild-type, *SPNS1*-KO, and *SPNS1*-AB cells grown in −choline or +choline medium. Data show means ± SEM (*n* = 3). (**D**) Representative fields from (C) at 72-hour time point. Green marks dead cells; red marks nuclei of live cells. Scale bar, 50 μm. (**E**) Schematic for subcutaneous engraftment of *SPNS1*-KO and *SPNS1*-AB PaTu-8988T tumors in mice. (**F**) Weights of dissected tumors after 62 days. Data show means ± SD (*SPNS1*-KO and *SPNS1*-AB in choline-deficient diet and *SPNS1*-KO in normal diet: *n* = 8; *SPNS1*-AB in normal diet: *n* = 10). Five mice were injected bilaterally per condition; tumors were omitted if mice died (two tumors each in SPNS1-KO in normal diet and SPNS1-AB in choline-deficient diete) or exhibited no engraftment (two tumors in *SPNS1*-KO in choline-deficient diet). Statistical test: Two-tailed unpaired *t* test. (**G**) Lethal fraction of wild-type, *SPNS1*-KO, and *SPNS1*-AB cells grown in serine-depleted medium (−serine) or medium supplemented with serine (27 mg/liter; +serine). Data show means ± SEM (*n* = 3). (**H**) Representative fields from (G) at the 72-hour time point. Green marks dead cells; red marks nuclei of live cells. Scale bar, 50 μm. (**I**) Live-cell abundance of PaTu-8988T KOs for cathepsin D (*CTSD*), *CTSB*, and *CTSL* and wild-type cells after culturing for 5 days in −choline or +choline medium. Data show means ± SEM (*n* = 3). Statistical test: Two-tailed unpaired *t* test. (**J**) Representative images of cells from (I) stained with propidium iodide (PI) for dead cells (PI pseudo-colored green for consistency with earlier panels). Scale bar, 50 μm. ns, not significant.

To further characterize how loss of *SPNS1* affects cell growth, we generated growth curves for PaTu-8988T *SPNS1*-KO cells under −choline and +choline conditions and compared these to the corresponding growth curves of wild-type and *SPNS1-*AB cells. To our surprise, cell growth was initially similar across the three different genotypes in +choline and −choline cultures (Fig. 2A). However, around 3 to 4 days in −choline medium, *SPNS1*-KO cell number declined markedly, while the wild-type and *SPNS1*-AB cells continued to increase in number. By day 6 in −choline medium, there was a significant decrease in the number of viable *SPNS1*-KO cells compared to wild-type and *SPNS1*-AB cells under the −choline condition (Fig. 2A). Decline in viable cell count was not observed under the +choline condition (Fig. 2A).

To verify that the decline in viable *SPNS1-*KO cell count resulted from cell death, we used the Incucyte system to monitor the lethal fraction of *SPNS1*-KO, wild-type, and *SPNS1-*AB cells over 5 days under +choline and −choline conditions (Fig. 2B) (*31*). Consistent with the growth curves, *SPNS1*-KO cells under the −choline, but not +choline, condition exhibited a remarkable, rapid loss of viability at approximately 3 days in culture (Fig. 2, C and D, and fig. S2, A and B). The cell death exhibited by *SPNS1*-KO cells preceded any loss in cell viability of wild-type cells under –choline condition by approximately 24 hours (Fig. 2, C and D, and fig. S2A) and was observed in a second PaTu-8988T *SPNS1*-KO cell line (fig. S2, C and D). Rapid cell viability loss was not observed under the +choline condition for any genotype (Fig. 2, C and D, and fig. S2B). Notably, reexpressing *SPNS1* in *SPNS1-*KO cells rescued their ability to survive choline deprivation to wild-type levels (Fig. 2, C and D, and fig. S2, A and B). Consistent with these results, we found that subcutaneous xenograft tumors developed from *SPNS1*-KO PaTu-8988T cells are significantly smaller than those from *SPNS1*-AB cells only in nonobese diabetic (NOD)/severe combined immunodeficient (scid) mice when fed a choline-deficient diet (Fig. 2, E and F).

Adaptation to metabolic stress is a major lysosomal function (*32*, *33*), and loss of *SPNS1* might have caused a general dysfunction that limited the ability of lysosomes to supply various nutrients including choline. To test that loss of cell viability in *SPNS1-*KO cells is specific to choline depletion, we generated lethal fraction curves for *SPNS1-*KO, wild-type, and *SPNS1-*AB PaTu-8988T cells in medium lacking serine, a nutrient that is similarly a constituent of polar lipid headgroups. While cell growth was slowed under the −serine condition across all genotypes, no *SPNS1*-dependent loss in cell viability was observed under −serine compared to +serine conditions (Fig. 2, G and H, and fig. S2, E and F). To further establish that the death of *SPNS1-*KO cells under −choline condition is due to a specific interaction between choline and *SPNS1* function, we tested PaTu-8988T cells deficient for the lysosomal proteases cathepsin D (CTSD), CTSB, and CTSL, whose loss causes lysosomal dysfunction and human diseases (*29*, *34*–*36*). Consistent with their role in maintaining lysosomal homeostasis, loss of these proteases leads to a general growth defect under +choline and −choline conditions compared to wild-type cells, with cells in −choline medium being slightly more affected in some cases (Fig. 2I). However, we did not observe any increased cell death in *CTSD-*KO, *CTSB-*KO, or *CTSL-*KO cells compared to wild-type cells growing in −choline medium (Fig. 2J). These results establish that SPNS1 supports cell survival under choline limitation through a function that is related to choline scavenging.

### SPNS1-deficient lysosomes accumulate lysophospholipids

On the basis of these results, and because SPNS1 localizes to the limiting lysosomal membrane and is structurally homologous to solute carriers in the major facilitator superfamily (*37*), we initially hypothesized that SPNS1 is a transporter of either choline or phosphocholine. These two molecules directly contribute to choline pools in the cytosol and their efflux from the lysosome has been established (*7*, *8*).

**Fig. 3.**
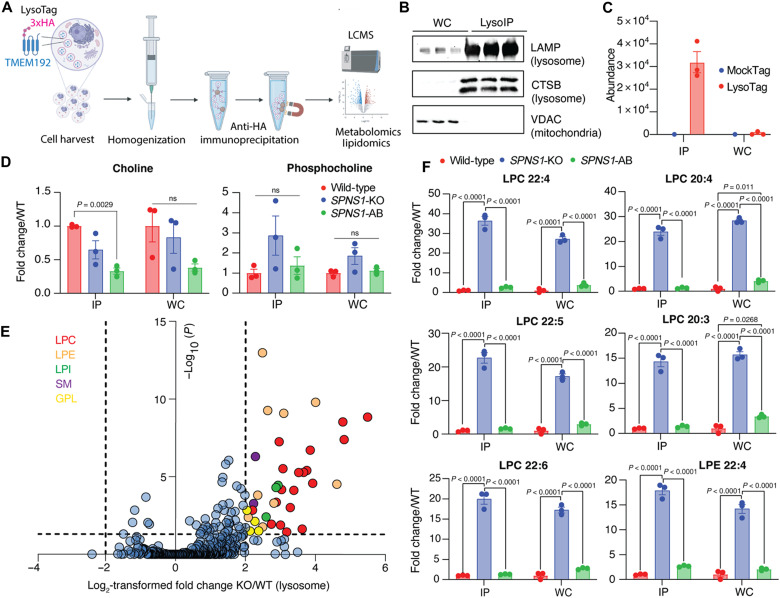
SPNS1-deficient lysosomes accumulate LPCs and LPEs. (**A**) Schematic of LysoIP workflow used in this study. (**B**) Immunoblot analysis for LysoIP validation on wild-type PaTu-8988T cells expressing LysoTag. Blots depict LAMP2, CTSB, and VDAC levels in whole-cell (WC) and LysoIP fractions for three biological replicates. (**C**) LysoTracker quantification in LysoIP (IP) and whole-cell fractions from wild-type PaTu-8988T cells expressing LysoTag (TMEM192-3xHA; *n* = 3; Data show means ± SEM) versus MockTag (TMEM192-3xflag; *n* = 1). (**D**) Quantitation of lysosomal (IP) and whole-cell abundance of choline and phosphocholine (normalized to a pool of endogenous amino acids: phenylalanine, methionine, and tyrosine in positive ion mode) in wild-type, *SPNS1*-KO, and *SPNS1*-AB cells. Data show mean of fold change versus wild-type ± SEM (*n* = 3). Statistical test: One-way ANOVA with Tukey’s post hoc test. ns, not significant. (**E**) Volcano plot presentation of untargeted lipidomics data [*SPNS1-*KO (KO) versus wild-type (WT)] highlighting LPCs (red), LPEs (orange), lysophosphatidylinositol (LPI; green), SM (purple), and glycerophospholipids (GPLs; yellow) (*n* = 3). One-way ANOVA with Tukey post hoc test was performed for *P* values. Adjusted *P* values were calculated by Benjamini-Hochberg correction for the false discovery rate at 5%. (**F**) Targeted quantitation of the top six accumulated LPCs and LPEs (normalized to internal standards from SPLASH LIPIDOMIX) in lysosomes (IP) and whole-cell fractions of wild-type, *SPNS1*-KO, and *SPNS1*-AB cells. Data show mean of fold change versus wild type ± SEM (*n* = 3). Statistical test: One-way ANOVA with Tukey’s post hoc test.

The substrate of a lysosomal transporter mediating efflux is predicted to accumulate in the lysosome upon loss of the transporter, as is observed in many human lysosomal storage diseases (*34*). Intralysosomal substrate accumulation can be robustly quantified using our rapid lysosomal immunopurification (LysoIP) method coupled with mass spectrometry (MS) (*3*, *4*, *38*). We first adapted our LysoIP pipeline to PaTu-8988T cells (Fig. 3A, Materials and Methods, and fig. S3A). We demonstrated efficient pull-down of PaTu-8988T lysosomes via capture of both lysosomal transmembrane and intraluminal proteins (Fig. 3B), as well as intralysosomal markers and metabolites (Fig. 3C and fig. S3B).

Unexpectedly, we observed no robust increase in the lysosomal levels of choline or phosphocholine upon *SPNS1* loss (Fig. 3D and fig. S3C). However, in the lysosomal metabolome derived from *SPNS1*-KO cells, we noticed striking elevations in features that were annotated as choline and phosphocholine based on mass/charge ratio (*m/z*) but eluted at early retention times corresponding to lipids (fig. S3D). We predicted that these must be the result of in-source fragmentation of choline- and phosphocholine-containing lipids. By reanalyzing our polar metabolite data to include lysophospholipids, we found massive accumulation of lipid catabolites tentatively annotated as LPCs and lysophosphatidylethanolamines (LPEs) in *SPNS1*-KO lysosomes (fig. S3E). These lysophospholipids are generated by the hydrolysis of one acyl group of their corresponding phospholipid. Of importance, the high abundance of LPCs and the matching retention time to that of the observed in-source fragments of choline and phosphocholine indicate that they are the source of detected features (fig. S3D).

While polar metabolite analysis can tentatively identify lysophospholipids, lipid analysis is needed to confirm their identity (*39*). We therefore performed unbiased lysosomal lipidomics and confirmed that LPCs and LPEs accumulate in lysosomes derived from *SPNS1-*KO cells (Fig. 3, E and F, and table S3), with conclusive molecular evidence at the tandem MS (MS/MS) level (fig. S3F). Targeted analyses further validated these results and demonstrated that reexpressing *SPNS1* completely rescued lysophospholipid accumulation (Fig. 3F). Because of the magnitude of the effect size in the lysosome, this accumulation is also observed at the whole-cell level (Fig. 3F). This result indicates that lysophospholipids are maintained at low levels in the cell, and an increase in their levels in the lysosome, despite its relatively small volume, can be detected at the whole-cell level. Last, both untargeted lipidomics (Fig. 3E) and targeted quantitation (fig. S3G) show that the level of sphingosine is not affected by the loss of SPNS1. This suggests that SPNS1 is not required for sphingosine efflux from lysosomes under our experimental conditions in PaTu-8988T cells (*30*). Together, our data support that SPNS1 is required for the clearance of LPCs and LPEs from the lysosome.

### SPNS1 exports LPCs from the lysosome for recycling to PCs in the cytosol

**Fig. 4.**
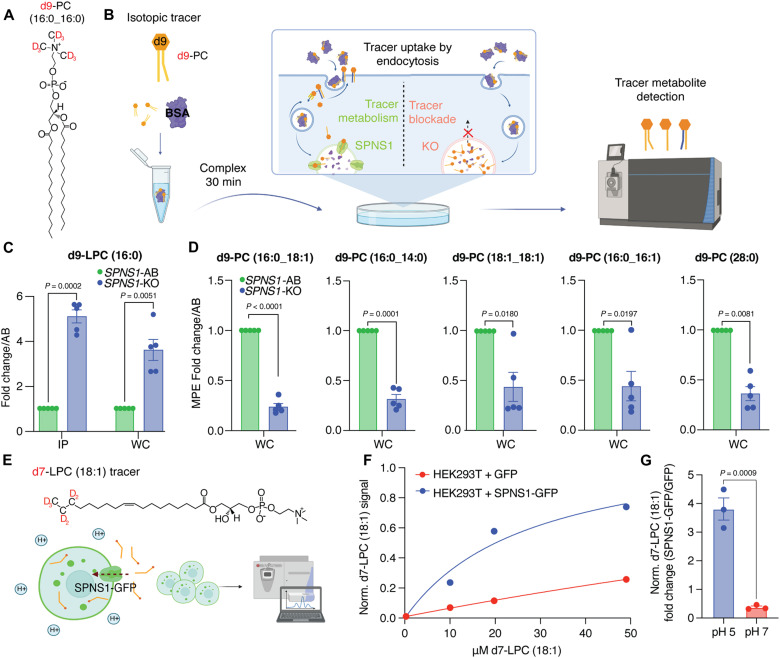
SPNS1 is the lysosomal transporter of LPC. (**A**) Structure of d9-PC (16:0) tracer used in this study. (**B**) Schematic of d9-PC (16:0) tracing assay used in (C) and (D). (**C**) Quantitation of d9-LPC (16:0) in the lysosomal and whole-cell fractions of *SPNS1*-KO and *SPNS1*-AB cells expressed as paired-sample fold change. Data show means ± SEM (*n* = 5). Statistical test: Two-tailed paired *t* test. (**D**) Quantitation of molar percent enrichment (MPE) for selected PCs expressed as paired-sample fold change. Data show means ± SEM (*n* = 5). Statistical test: Two-tailed paired *t* test. (**E**) Schematic of d7-LPC (18:1) surface transport assay and d7-LPC (18:1) structure. (**F**) Dose-response curve for d7-LPC (18:1) transport in HEK293T cells overexpressing GFP or SPNS1-GFP. Data show d7-LPC (18:1) signal normalized to that of the endogenous lipid PC(16:0_18:0) (POPC). Assay was performed at pH 5 on the outside. (**G**) d7-LPC (18:1) transport in HEK293T cells overexpressing GFP or SPNS1-GFP at acidic pH (pH 5) and neutral pH (pH 7) using 20 μM substrate. Data are presented as fold change of d7-LPC (18:1) signal in HEK293T + SPNS1-GFP over HEK293T + GFP. Data show means ± SEM (*n* = 3). Statistical test: Two-tailed unpaired *t* test.

On the basis of our observations, we proposed that SPNS1 is a lysosomal lysophospholipid transporter. To test this hypothesis, we focused on LPC due to (i) its direct involvement in choline metabolism and (ii) its extreme accumulation in *SPNS1*-KO lysosomes. First, we leveraged isotope tracing [as previously described (*38*, *40*)] to assess whether LPC generated from lysosome-targeted deuterated PC is trapped in the lysosomes of SPNS1-deficient cells (Fig. 4, A and B). After verifying equal tracer uptake by *SPNS1*-KO and *SPNS1*-AB cells (fig. S4A), we found a robust fivefold accumulation of deuterated LPC in SPNS1-deficient lysosomes, supporting the putative role of SPNS1 in lysophospholipid efflux (Fig. 4C). Second, we assessed the abundance of labeled PCs in the whole-cell fractions of *SPNS1*-KO and *SPNS1*-AB cells, as LPCs transported from lysosomes are expected to contribute to PC pools in the cytoplasm upon re-esterification via the Lands Cycle (*41*, *42*). Using whole-cell isotope tracing, we found that SPNS1 is required for LPC generated in the lysosome to contribute to PC pools in the cytoplasm, presumably by enabling LPC egress from lysosomes (Fig. 4D). Of importance to this assumption, loss of SPNS1 did not reduce basal choline phospholipid biosynthesis or turnover, as similar or higher labeling of PC species was observed in *SPNS1*-KO compared to *SPNS1*-AB cells when free d9-choline was used as a tracer (fig. S4B).

Last, to provide definitive evidence for the role of SPNS1 in LPC transport, we leveraged the overexpression system of SPNS1–green fluorescent protein (GFP) in human embryonic kidney (HEK) 293T cells, which leads to spilling over of SPNS1-GFP to the plasma membrane (fig. S5A). Using a cell-based uptake assay (Fig. 4E), we found that SPNS1-GFP mediates a robust transport of LPC (Fig. 4F) in a pH-dependent manner (Fig. 4G), consistent with its role as lysosomal transporter of lysophospholipids.

### SPNS1-dependent LPC efflux from the lysosome is required for cell survival under choline deprivation

**Fig. 5.**
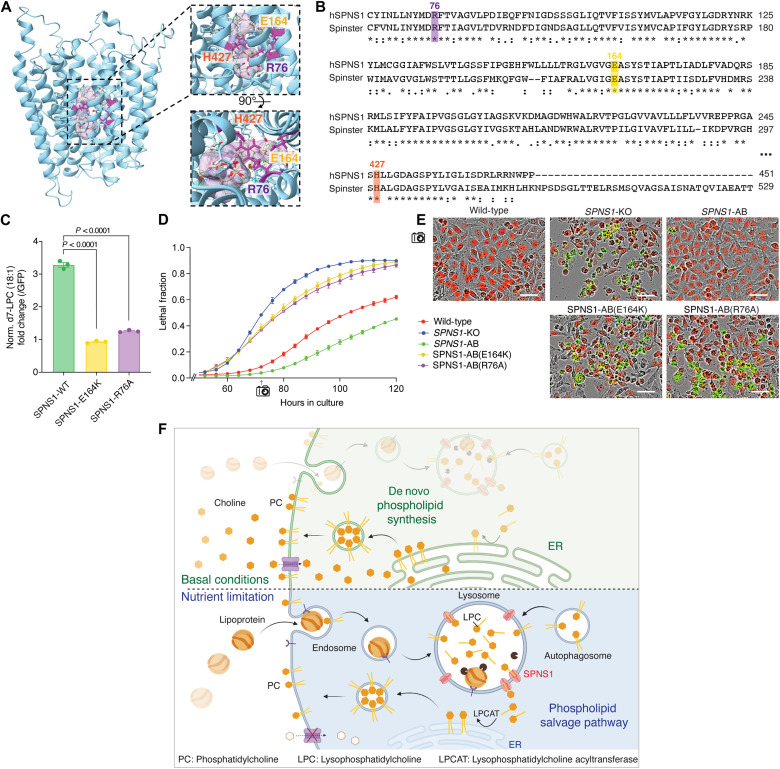
LPC efflux from lysosomes mediates cell survival under choline limitation. (**A**) Schrödinger Glide–based docking of LPC (22:4) onto to the predicted human SPNS1 I-TASSER structure indicates that conserved residues, including R76, H427, and E164, may be involved in LPC (22:4) stabilization and transport. (**B**) Multiple sequence alignment between human SPNS1 and *D. melanogaster* homolog Spinster indicates the presence of fully conserved residues (marked with asterisks). The conserved glutamic acid residue responsible for loss-of-function mutations in Spinster, E217, corresponds to E164 in human SPNS1 (highlighted in yellow). (**C**) d7-LPC (18:1) transport in HEK293T cells overexpressing SPNS1-GFP, SPNS1(E164K)-GFP, or SPNS1(R76A)-GFP. Data are presented as fold change of d7-LPC (18:1) signal in HEK293T expressing wild-type or mutant SPNS1-GFP over HEK293T expressing soluble GFP. The assay was performed with 20 μM d7-LPC (18:1) at pH 5. Data show means ± SEM (*n* = 3). Statistical test: Two-tailed unpaired *t* test. (**D**) Lethal fraction of wild-type, *SPNS1*-KO, *SPNS1*-AB, and *SPNS1*-AB mutants grown in choline-depleted (−choline) medium. Data show means ± SEM (*n* = 3). (**E**) Representative Incucyte fields from (D) at the 72-hour time point. Green marks dead cells; red marks nuclei of live cells. Scale bars, 50 μm. (**F**) Model for a lysosomal lipid salvage pathway in which SPNS1 transports LPC to the cytosol for reacylation to PC. The pathway becomes essential for cell survival under choline-limited conditions when substrates for de novo PC synthesis are restricted.

To test whether lysophospholipid efflux from the lysosome is the critical function of SPNS1 that mediates cell survival under choline deprivation, we sought to identify SPNS1 mutants defective in transporting LPC. We predicted SPNS1 structure using Iterative Threading ASSembly Refinement (I-TASSER) and identified the putative substrate binding pocket in the core of the protein (Fig. 5A). Notably, this pocket is in close proximity to E164, corresponding to E217 in the *Drosophila melanogaster* homolog Spinster, a missense point mutation (E217K) in which causes a lysosome storage disease-like phenotype in flies (*10*). Reasoning E164 is thus likely to be critical for substrate binding, we used it to guide docking of LPC (22:4) to the putative substrate binding pocket using Schrödinger Glide (Fig. 5A). From this model, we identified the conserved residues R76 and H427 as additional key residues that contact the LPC substrate and mediate stabilization, with R76 in close proximity to, and predicted to form a salt bridge with E164 (Fig. 5, A and B). For follow-up studies, we mutated E164 to lysine and R76 to alanine to generate mutants SPNS1(E164K) and SPNS1(R76A), respectively. Consistent with structural predictions, both mutations abrogated the transport function of SPNS1 in cell-based uptake assays (Fig. 5C), while their expression on plasma membrane was comparable to that of wild type (fig. S5A). Next, we tested whether SPNS1 transport-defective mutants were capable of rescuing *SPNS1*-KO cell death under choline limitation. We first expressed SPNS1 mutants in *SPNS1*-KO cells to generate *SPNS1*(E164K)-AB and *SPNS1*(R76A)-AB cells and validated the proper lysosomal localization of these mutants (fig. S5B). We then monitored the lethal fraction of each group in −choline and +choline medium using the Incucyte system. Expectedly, SPNS1(E164K) and SPNS1(R76A) were not able to rescue the cell death under −choline condition to the level of wild-type SPNS1 (Fig. 5, D and E, and fig. S5, C and D). No cell death was observed under the +choline condition for any genotype (fig. S5, E and F). These results indicate that LPC efflux from the lysosome is the key SPNS1 function required for cell survival under choline deprivation.

## DISCUSSION

Our work identifies the orphan transmembrane protein SPNS1 as a transporter for LPC (and putatively, LPE) that is necessary for lysosomal phospholipid salvage and recycling. SPNS1-transported LPCs support phospholipid biosynthesis in the cytosol and become essential when exogenous choline for de novo synthesis of phospholipids is limited (Fig. 5F).

Biochemical studies have previously established that lysosomes recycle choline-related catabolites derived from the degradation of PC and SM (*7*, *8*). Seminal experiments by Fowler and De Duve (*7*) demonstrated that rat liver lysosomal extracts hydrolyze phospholipids to liberate glycerophosphodiesters (GPDs), including glycerophosphocholine (GPC) as terminal catabolites. Our group recently found that GPDs are exported from the lysosome in a CLN3-dependent manner (*38*). Similarly, early work using radiolabeled choline tracers revealed that SM is hydrolyzed to ceramide and phosphocholine by the lysosomal acid sphingomyelinase. Phosphocholine then exits the lysosome through a yet unidentified transporter (*8*). Once GPC and phosphocholine reach the cytosol, they are used for the synthesis of PCs and SMs (*5*, *8*, *38*). The biochemical mechanism proposed to explain these observations has been that GPDs and phosphocholine are the terminal choline-containing catabolites exported from the lysosome (*7*).

While we initially set out to identify the elusive phosphocholine transporter, our data provide strong evidence that an active machinery to transport LPC, and potentially the closely related lipid LPE, from lysosomes also exists. LPC and LPE are formed by the monodeacylation of PC and PE, respectively, and are themselves deacylated once more to produce the dideacylated catabolites GPC and glycerophosphoethanolamine (GPE), respectively (*7*). Lysophospholipid export from the lysosome therefore prevents phospholipid catabolism to the terminal GPC and GPE catabolites, salvaging lysophospholipid intermediates for direct reacylation to various PC species—a stratagem that is easily rationalized as conserving energy equivalents that would otherwise be consumed in a futile deacylation-reacylation cycle between LPC/GPC and LPE/GPE. In support of this model, our isotope tracing establishes that salvaged LPC is reacylated to PC upon export to the cytosol, likely via a one-step re-esterification mechanism using the endoplasmic reticulum (ER)–resident enzyme LPC acyltransferase (LPCAT) (*41*). Lysophospholipid salvage is a mechanism that can sustain phospholipid synthesis under conditions of nutrient limitation. Consistent with this, we establish that LPC transport by SPNS1 is essential under choline deprivation.

It is additionally plausible that SPNS1-supplied lysosomal LPCs have a dedicated cellular function. Studies have implicated *SPNS1* in autophagosome formation (*43*), a process requiring PC synthesis for membrane nucleation (*44*). Depletion of SPNS1 protein leads to defective autophagic lysosome reformation, ultimately affecting autophagy-dependent processes such as synaptic pruning and neurodevelopment (*45*). Hence, it is possible that SPNS1-supplied lysosomal LPCs are shunted toward biosynthesis of PC specifically for autophagosome membranes. Lysophospholipids are also themselves functional, bioactive signaling molecules that mediate a variety of cellular responses via specific G protein–coupled receptors (*46*). Lysosomal LPC could function as intracellular signaling messenger, consistent with the emerging role of lysosomes as signaling organelles.

While prior studies have variably suggested SPNS1 involvement in lysosomal transport of carbohydrates (*9*) or sphingolipids (*30*), we find that SPNS1 is a pH-dependent lysosomal transporter of lysophospholipids. Notably, our observations are consistent with very recent work by a number of groups, including (i) He *et al.* (*47*) that described lysophospholipid transport by SPNS1; (ii) Dastvan *et al.* (*48*) that described a proton-driven, alternating-access mechanism for SPNS transporters; and (iii) Zhou *et al.* (*30*) that predicted a lysolipid-like transport substrate for SPNS1 based on structural modeling of a bacterial homolog. Moreover, our work provides a notable advance in understanding the physiological role of SPNS1-mediated lysophospholipid transport by demonstrating its critical importance under conditions of nutrient limitation.

Advancing our understanding of *SPNS1* function could ultimately have important implications for human disease. Defects in lysosomal lipid catabolic pathways and lysosomal transporters are known causes of severe, early-onset, and age-related neurodegenerative diseases (*29*, *49*). For instance, deficiencies in acid sphingomyelinase (catalyzing lysosomal SM hydrolysis) and NPC1 and CLN3 (lysosomal lipid or lipid-derived metabolite transporters) cause pathologic accumulation of lysosomal storage materials and present clinically as lysosomal storage diseases with early-onset and fatal neurodegeneration (*29*). To date, *SPNS1* has not been identified as a genetic cause of a neurodegenerative disorder in humans. Nevertheless, deficiency in *SPNS1*’s orthologs has been shown to lead to neurodegeneration in flies (*10*) and to lysosomal dysfunction and storage phenotypes in zebrafish models (*43*). Thus, while germline loss-of-function *SPNS1* mutations might not be compatible with mammalian life, *SPNS1* deficiency in cells and model organisms represents a model for lysosomal lipid storage and dysfunction of potential relevance to the study of lysosome-related pathophysiologic mechanisms of neurodegenerative disease.

*SPNS1* has also been identified as a genetic risk factor for various health disorders. Notably, it was found to be a negative prognostic marker in cancer (*50*, *51*). Because choline is highly enriched in rapidly proliferating tumors that require it for lipid biosynthesis, it follows that cancer cells might induce *SPNS1* expression as a cellular strategy to salvage phospholipids for biomass production. *SPNS1* was additionally found to be a host factor facilitating severe acute respiratory syndrome coronavirus 2 virus entry into lung tissue, possibly through supplying lysophospholipids that enhance membrane fusion (*52*). SPNS1 could therefore be a candidate therapeutic target in cancer and the treatment of viral illnesses. Ultimately, our understanding of the biochemical and physiologic functions of SPNS1 is still in the nascent stages, and follow-up investigations are needed to characterize the pathophysiologic connections and study the implications of perturbing lysophospholipid efflux through SPNS1 in physiology and disease.

In summary, through uncovering SPNS1 and its role as an LPC transporter in a lysosomal phospholipid salvage pathway, our work demonstrates the power of our platform that combines endolysosomal CRISPR-Cas9 screens with engineered lysosomal dependencies to uncover the biochemical functions of orphan lysosomal proteins and previously undiscovered metabolic pathways.

## MATERIALS AND METHODS

### Cell culture

Unless otherwise indicated, all cell lines and their derivatives were cultured in Dulbecco’s modified Eagle’s medium (DMEM; Gibco) supplemented with 10% heat-inactivated fetal bovine serum (FBS) (Thermo Fisher Scientific), GlutaMAX (Gibco), and penicillin and streptomycin (Thermo Fisher Scientific). For studies using nutrient-depleted medium, custom medium was prepared. Choline-depleted (−choline) medium consisted of 1:1 DMEM/Ham’s F12 without sodium bicarbonate, serine, methionine, or choline chloride (Caisson Laboratories, DFP14-5) supplemented with sodium bicarbonate (1200 mg/liter), methionine (18 mg/liter), serine (27 mg/liter), 5% triple-dialyzed FBS, and penicillin/streptomycin. To make +choline medium, −choline medium was supplemented with 100 μM choline chloride. Serine-depleted medium consisted of 1:1 DMEM/Ham’s F12 without sodium bicarbonate, serine, methionine, or choline chloride (Caisson Laboratories; DFP14-5) supplemented with sodium bicarbonate (1200 mg/liter), methionine (18 mg/liter), 100 μM choline chloride, 5% triple-dialyzed FBS, and penicillin/streptomycin. To make +serine control medium, −serine medium was supplemented with serine (27 mg/ml).

All cell lines were maintained at 37°C and 5% CO_2_. All cell lines were frequently tested for mycoplasma. The following cell lines were used in this study: PaTu-8988T (human, RRID no. CVCL_1847) and A549 (human, RRID no. CVCL_0023).

### Recombinant virus production and transduction

#### Production

Viral HEK293T cells were grown to 40 to 60% confluency and cotransfected with lentiviral or retroviral packaging plasmid, vesicular stomatitis virus glycoprotein (VSV-G) pseudotyping plasmid, and vector (or vector library) plasmid at a mass ratio of 9:1.5:10, respectively, using the XtremeGene 9 (Roche) transfection reagent. Sixteen hours after transfection, culture medium was replaced with DMEM supplemented with 30% FBS and penicillin/streptomycin. The virus-containing supernatant was harvested after 48 hours (first collection) and 96 hours (second collection), centrifuged to remove cell debris, and filtered through a 0.22-μm syringe filter.

#### Transduction

Two million cells were plated in six-well plates in DMEM with the addition of polybrene (8 μg/ml) and 100 to 250 μl of virus-containing medium. Spin infection was performed at 2200 rpm for 45 min at 37°C, and cells were incubated with virus for 16 hours before adding fresh culture medium containing the relevant antibiotic for selection for at least 72 hours.

### Endolysosomal CRISPR-Cas9 screen

#### Endolysosomal library construction

A list of lysosomal, endocytic, and autophagy-related genes in addition to a selective set of metabolism and signaling genes (1061 genes) was manually curated by unbiased profiling of lysosomal proteins using in-house LysoIP proteomics and by selecting genes implicated in endolysosomal trafficking (*53*). For the library design, 10 targeting sgRNAs were selected per gene, and an additional 1050 sgRNAs (~9% of the library) were included as safe-targeting (control) sgRNAs. These guides were cloned into pMCB320 as previously described (*54*).

#### Endolysosomal screen

The endolysosomal library was infected into Cas9-expressing PaTu-8988T pancreatic cancer cells at 1000× library coverage and selected using puromycin. At time point 0, cells were trypsinized, washed, and split into two culture medium conditions: choline-depleted medium (−choline) or choline-supplemented medium (+choline). Choline-depleted medium was composed of 1:1 DMEM/Ham’s F12 without sodium bicarbonate, serine, methionine, or choline chloride (DFP14-5, Caisson Laboratories) supplemented with sodium bicarbonate (1200 mg/liter), methionine (18 mg/liter), serine (27 mg/liter), 5% triple-dialyzed FBS, and penicillin/streptomycin. To make +choline medium, −choline medium was supplemented with 100 μM choline chloride.

Cells were propagated in −choline or +choline medium for 14 doublings, while maintaining a library coverage of >1000×. DNA from time point 0 and after 14 doublings for each condition was isolated, and sgRNA sequences were polymerase chain reaction (PCR)–amplified. Illumina universal adaptor sequences were subsequently added to sgRNA amplicons by PCR, and sgRNA abundances were determined using the Illumina NextSeq 550 with NextSeq 500/550 mid-output kit v2.5.

#### Screen analysis

Sequencing data were analyzed using the CasTLE method developed by Morgens *et al.* (*17*). Briefly, the method calculates the most likely maximum effect (phenotype) size (CasTLE effect) among each group of gene-targeting sgRNAs by comparing each set to the negative sgRNAs (control and safe targeting) in the library. The method then scores the significance of the effect (CasTLE score) by permuting the results.

### Generation of KO cell lines

The following *SPNS1* sgRNAs were used in this study: *SPNS1*-sg1, 5′-GTCGGCCACAAAGAGGT-3′; *SPNS1*-sg2, 5′-GAAGTATCTCATGTGCGG-3′. *SPNS1*-targeting sgRNAs were cloned into the PX458 vector containing GFP as a selectable marker. sgRNA-PX458 plasmids were transfected into cells using XtremeGene 9 transfection reagent, and 48 hours after transfection, GFP^+^ cells were single-sorted into 96-well plates prepared with DMEM (200 μl per well) with 30% FBS and penicillin/streptomycin. Plates were incubated at 37°C for 3 weeks at 5% CO_2_ and routinely checked for colony formation. Colonies were harvested and sgRNA target sites PCR-amplified and Sanger-sequenced. Indels generated at the sgRNA target site were determined using Synthego ICE indel deconvolution web-based tool (https://synthego.com). *CTSB*, *CTSD*, and *CTSL* KO lines were generated using pLentiCRISPRv1 and previously validated (*55*).

### Screen hits validation by end point viability

Screen hits were validated by generating KO cell lines and comparing viability between cells cultured in −choline and +choline medium after 5 days. Cells were seeded in triplicate in 96-well opaque-edge clear-bottom plates (Corning) at a density of 2000 cells per well and incubated at 37°C and 5% CO_2_ until the most confluent wells reached ~95% confluence (approximately 5 days) without medium change. Cell viability was measured using the CellTiter-Glo luminescent viability assay (Promega) on a SpectraMax i3 plate reader (Molecular Devices).

### Incucyte live-cell analysis and lethal fraction calculations

Lethal fraction curves were produced using an Incucyte S3 (Sartorious) as previously described (*31*). Briefly, live cells were labeled with nuclear-localized red fluorescent protein mKate2, and the frequency of live cells was determined by counting mKate2^+^ (red) nuclei per field; dead cells were labeled using SYTOX green (SG) viability dye (Invitrogen), and the frequency of dead cells was determined by counting green cells per field. mKate2^+^ and SG^+^ (double-positive) cells detected in the overlap channel were defined as dead cells (recently dead cells may stain double positive due to residual mKate2 signal). Lethal fraction (LF) was calculated as $1-\frac{\mathrm{mkate}2^{+}}{\mathrm{mKate}2^{+}+\mathrm{MAX}[{\mathrm{SG}}^{+}]}$, where mKate2^+^ cells exclude double-positive mKate2^+^SG^+^ cells and MAX[SG^+^] is the maximum number of SG^+^ cells observed in the given field at any analysis time point (the MAX[SG^+^] metric accounts for the disappearance of dead cells over time). Incucyte analysis parameters were optimized for the detection of live and dead cells for the PaTu-8988T cell type. Parameters are indicated below and were kept consistent across all lethal fraction analyses.

For green parameters, in segmentation, selection = adaptive and threshold = 1.5 green calibrated units; in edge sensitivity, selection = edge-split on and sensitivity = −30; and in filters, selection = area and range = 50 to 750 μm. For red parameters, in segmentation, selection = top-hat, radius = 100, and threshold = 3.0 red calibrated units; in edge sensitivity, selection = edge-split on and sensitivity = −10; and in filters, selection = area and range = 50 to 1000 μm.

For cell lines not infected with mKate2 red fluorescent nuclear protein, cell death was assessed using propidium iodide (PI) viability stain. The Incucyte system was used to image PI staining over time or at one end point.

### Cell growth curves

Cell growth curves were generated by assessing live-cell abundance using CellTiter-Glo Luminescent Cell Viability Assay (Promega). Cells were seeded in triplicate at a density of 2000 cells per well in seven separate opaque-walled plates. One plate was used for viable cell analysis by CellTiter-Glo at each time point to generate a time-dependent growth curve.

### Immunoblotting

Cell lysates were resolved by SDS–polyacrylamide gel electrophoresis (Thermo Fisher Scientific) at 80 V and transferred to polyvinylidene difluoride membranes for 2 hours at 40 V. Membranes were blocked with 5% BSA (bovine serum albumin) in TBST buffer (tris-buffered saline with Tween 20) for 30 min and then incubated overnight with primary antibodies in 5% BSA in TBST at 4°C. The following primary antibody dilutions were used: lysosome-associated membrane protein 2 (LAMP2) (1:1000; H4B4, Santa Cruz Biotechnology, RRID: AB_626858), voltage-dependent anion channel (VDAC) (1:1000; B-6, Santa Cruz Biotechnology, RRID: AB_2750920), and Cathepsin B (CTSB) (1:1000; D1C7Y, Cell Signaling Technology, RRID: AB_2687580). After incubation, membranes were washed three times with TBST for 15 min per wash and incubated with the appropriate horseradish peroxidase–linked secondary antibodies (Cell Signaling Technology, RRIDs: AB_330924 and AB_2099233) diluted 1:3000 in 5% BSA in TBST for 1 hour at room temperature. Last, membranes were washed three times with TBST and visualized using ECL2 Western blotting substrate (Thermo Fisher Scientific) on a ChemiDoc MP imaging system (Bio-Rad).

### Immunofluorescence

Cells plated on glass coverslips were fixed with 4% paraformaldehyde and blocked with 3% BSA in phosphate-buffered saline (PBS) for 1 hour at room temperature. Primary antibodies were incubated overnight at 4°C with dilutions as follows: mouse anti-LAMP2 (1:500; H4B4, Santa Cruz Biotechnology, RRID: AB_626858) and rabbit anti–hemagglutinin (HA) (1:500; C29F4, Cell Signaling Technology, RRID: AB_1549589). Secondary antibodies (Alexa Fluor) were then applied in 1% BSA and 0.3% Triton X-100 for 3 hours. The following secondary antibodies were used (1:1000): goat anti-mouse Alexa Fluor 647 (A-21240, Thermo Fisher Scientific, RRID: AB_2535809) and goat anti-rabbit Alexa Fluor 594 (A32740, Thermo Fisher Scientific, RRID: AB_2762824). Confocal images were acquired on the Zeiss LSM980 with Airyscan 2.

### Cell volume measurement

Cell volumes were determined using a Beckman Z2 particle counter and size analyzer with filtering criteria set to include cells between 10 and 30 μm.

### Lysosomal immunopurification

Lysosome purification by LysoIP (DOI: dx.doi.org/10.17504/protocols.io.bybjpskn) was adapted from the method previously described (*4*) and optimized for the PaTu-8988T cell line. Because we observed better enrichment after optimization in *SPNS1*-KO clone #2 compared to clone #1, we used clone #2 for all main figure LysoIP and tracing experiments. Similar LysoIP results were obtained with the other clone. Briefly, cells were grown to 95% confluence in 15-cm cell culture dishes. If lysosomal isolates were to be used for downstream metabolomics analysis, 4 μM LysoTracker Red DND-99 (Invitrogen) was added to each plate 1 hour before cell harvest. At the time of harvest, each plate was washed 2× with 5 ml of ice-cold PBS, and cells were scraped in 1 ml of ice-cold KPBS [136 mM KCl and 10 mM KH_2_PO_4_ (pH 7.25) in Optima liquid chromatography (LC)–MS water] and transferred to a 2-ml tube. Cells were pelleted by centrifugation at 1000*g* for 2 min at 4°C. Following centrifugation, cells were resuspended in 500 μl of fresh KPBS, and 12.5 μl of the cell suspension was taken for whole-cell fraction analysis. The remaining cell suspension was lysed by trituration using a 29.5-gauge insulin syringe (EXELINT international), then diluted with an additional 500 μl of KPBS, and centrifuged at 1000*g* for 2 min at 4°C to pellet cell debris. The lysosome-containing supernatant (1 ml) was added to tubes containing an equivalent of 100 μl of Pierce anti-HA magnetic beads (Thermo Fisher Scientific) and rocked for 3 min at 4°C. Following incubation, the bead-lysosomes complexes were washed 3× with ice-cold KPBS and extracted in the appropriate buffer. For metabolite extraction, lysosome and whole-cell fractions were extracted in 50 and 225 μl, respectively, of 80% methanol in LC-MS water containing 500 nM isotope-labeled amino acids used as internal standards (Cambridge Isotope Laboratories). Following extraction, HA-binding beads were removed from the lysosome fractions using a rotary magnet, and cell debris was removed from whole-cell extractions by centrifugation at 20,000*g* at 4°C for 15 min. For lipidomic extraction, lysosome and whole-cell fractions were extracted in 1 ml of chloroform:methanol at ratio of 2:1 (v/v) containing SPLASH LipodoMix internal standard mix (750 ng/ml; Avanti) for >10 min. Following extraction, HA-binding beads were removed using a rotary magnet, and LysoIP and whole-cell samples were vortexed for 1 hour at 4°C. Two hundred microliters of saline was then added to each sample, and samples were vortexed for an additional 10 min. Vortexed samples were spun at 3000*g* for 5 min to separate polar (top) and nonpolar (bottom) phases. Six hundred microliters of the lipid-containing chloroform (nonpolar) phase was dried and reconstituted in acetonitrile:isopropanol:water of 13:6:1 (v/v/v). Both metabolomic and lipidomic extractions were analyzed on mass spectrometer with workflow and parameters described below.

### Untargeted metabolomics

Profiling of polar metabolites was performed on an ID-X Tribrid mass spectrometer (Thermo Fisher Scientific) with an electrospray ionization (ESI) probe. A SeQuant ZIC-pHILIC 150-mm by 2.1-mm column (1504600001, MilliporeSigma) coupled with a 20-mm by 2.1-mm (1504380001, MilliporeSigma) guard was used to carry out hydrophilic interaction chromatography (HILIC) for metabolite separation before MS. Mobile phases: A, 20 mM ammonium carbonate and 0.1% ammonium hydroxide dissolved in 100% LC-MS grade water; B, 100% LC-MS grade acetonitrile. Chromatographic gradient: linear decrease from 80 to 20% B from 0 to 20 min; fast linear increase from 20 to 80% B from 20 to 20.5 min; 80% B hold from 20.5 to 29.5 min. Flow rate, 0.15 ml/min. Injection volume, 1.5 to 2.5 μl. Mass spectrometer parameters: ion transfer tube temperature, 275°C; vaporizer temperature, 350°C; Orbitrap resolution, 120,000; radio frequency (RF) lens, 40%; maximum injection time, 80 ms; automatic gain control (AGC) target, 1 × 10^6^; positive ion voltage, 3000 V; negative ion voltage, 2500 V; Aux gas, 15 U; sheath gas, 40 U; sweep gas, 1 Arb. Full scan mode with polarity switching at *m*/*z* 70 to 1000 was performed. EasyIC was used for internal calibration. For data-dependent MS2 collection, pooled samples were prepared by combining replicates. Higher-energy collisional dissociation (HCD) energies, 15, 30, and 45%; AGC target, 2 × 10^6^; Orbitrap resolution, 240,000; maximum injection time, 100 ms; isolation window, 1 *m*/*z*; intensity threshold, 2 × 10^4^; exclusion duration, 5 s. Isotope exclusion was enabled. Background exclusion was performed via AcquireX with one header blank and the exclusion override factor set to 3.

Rigorous quantification of metabolite abundance was performed by TraceFinder (Thermo Fisher Scientific) in conjunction with an in-house library of known metabolite standards [Mass Spectrometry Metabolite Library (MSMLS), Sigma-Aldrich]. Mass tolerance for extracting ion chromatograms is 5 parts per million (ppm).

### Untargeted lipidomics workflow

Profiling of nonpolar lipids was performed on an ID-X Tribrid mass spectrometer (Thermo Fisher Scientific) with a heated ESI probe. An Ascentis Express C18 150-mm by 2.1-mm column (53825-U, MilliporeSigma) coupled with a 5-mm by 2.1-mm guard (53500-U, Sigma-Aldrich) was used to carry out C18-based lipid separation before MS. Mobile phases: A, 10 mM ammonium formate and 0.1% formic acid dissolved in 60 and 40% LC-MS grade water and acetonitrile, respectively; B, 10 mM ammonium formate and 0.1% formic acid dissolved in 90 and 10% LC-MS grade 2-propanol and acetonitrile, respectively. Chromatographic gradient: isocratic elution at 32% B from 0 to 1.5 min; linear increase from 32 to 45% B from 1.5 to 4 min; linear increase from 45 to 52% B from 4 to 5 min; linear increase from 52 to 58% B from 5 to 8 min; linear increase from 58 to 66% B from 8 to 11 min; linear increase from 66 to 70% B from 11 to 14 min; linear increase from 70 to 75% B from 14 to 18 min; linear increase from 75 to 97% B from 18 to 21 min; hold at 97% B from 21 to 35 min; linear decrease from 97 to 32% B from 35 to 35.1 min; hold at 32% B from 35.1 to 40 min. Flow rate, 0.26 ml/min. Injection volume, 2 to 4 μl. Column temperature, 55°C. Mass spectrometer parameters: ion transfer tube temperature, 300°C; vaporizer temperature, 375°C; Orbitrap resolution, MS1: 120,000; MS2: 30,000; RF lens, 40%; maximum injection time MS1: 50 ms and MS2: 54 ms; AGC target MS1, 4 × 10^5^ and MS2, 5 ×10^4^; positive ion voltage, 3250 V; negative ion voltage, 3000 V; Aux gas, 10 U; sheath gas, 40 U; sweep gas, 1 Arb. HCD fragmentation, stepped 15, 25, and 35%; data-dependent MS/MS (ddMS2) cycle time, 1.5 s; isolation window, 1 *m*/*z*; microscans, 1 U; intensity threshold, 1.0 ×10^4^; dynamic exclusion time, 2.5 s. Isotope exclusion was enabled. Full scan mode with ddMS2 at *m*/*z* of 250 to 1500 was performed. EasyIC was used for internal calibration. LipidSearch and Compound Discoverer (Thermo Fisher Scientific) were used for unbiased differential analysis. Lipid annotation was acquired from LipidSearch with the precursor tolerance at 5 ppm and the product tolerance at 8 ppm. The mass list was then exported and used in Compound Discoverer for improved alignment and quantitation. Mass tolerance, 10 ppm; minimum and maximum precursor mass, 0 to 5,000 Da; retention time limit, 0.1 to 30 min; peak filter signal-to-noise ratio, 1.5; retention time alignment maximum shift, 1 min; minimum peak intensity, 10,000. Compound detection signal-to-noise ratio was set to 3. Isotope and adduct settings were kept at default values. Gap filling and background filtering were performed by default settings. The MassList Search was customized with 5-ppm mass tolerance and 1-min retention time tolerance. Area normalization was performed by constant median after blank exclusion.

### ^2^H-isotope tracing by d9-dipalmitoylphosphatidylcholine (16:0_16:0) in PaTu-8988T cells

*SPNS1*-KO and *SPNS1-*AB PaTu-8988T cells were seeded according to LysoIP procedure. Upon ~95% confluency, the cells were washed by PBS, and the medium was replaced with FBS-depleted medium for 3 hours for serum starvation. The cells were subsequently labeled with 37.6 μM d9-dipalmitoylphosphatidylcholine (16:0_16:0) conjugated with 0.25% fatty acid–free BSA for 2 hours. Whole-cell and lysosomal fractions were obtained according to the LysoIP procedure.

To assess lysosomal contribution to choline-containing lipids at the whole-cell level, molar percent enrichment (MPE) for trimethyl-d9 labeling was calculated on the basis of the following equation

$$\mathrm{MPE}=\frac{\sum_{i=0}^{n}i\times m_{i}}{n\times \sum_{i=0}^{n}\;m_{i}}\times 100\%$$

where 𝑛 is the number of hydrogen atoms in the lipid molecule, 𝑚𝑖 the abundance of a mass isotopomer, and 𝑖 the labeling state (M + *i*). As a choline-containing lipid can only be present as either unlabeled (M + 0) or d9-labeled (M + 9), the equation above is simplified as follows

$$\mathrm{MPE}=\frac{d9-\text{labeled speclies}}{d9-\text{labeled species}+\text{unlabeled species}}\times 100\%$$

### ^2^H-isotope tracing by d9-choline in PaTu-8988T cells

*SPNS1*-KO and *SPNS1*-AB PaTu-8988T cells were seeded at 1.5 million per well in six-well plates. Upon full confluency, the cells were first washed by PBS and then labeled with 37.6 μM d9-choline chloride mixed with 0.25% fatty acid–free BSA for 3 hours. The whole-cell harvest was then processed for total lipid analysis. MPE was calculated as mentioned previously.

### Transport assay

Equal numbers of HEK293T cells expressing soluble GFP or overexpressing GFP fusions to SPNS1 or mutant SPNS1 were incubated in 100 μl of transport assay buffer with the indicated concentration of d7-LPC (18:1) (Avanti) for 20 min at 37°C. The composition of transport assay buffers was as follows: pH 5: 25 mM sodium acetate, 5 mM glucose, 1 mM MgCl, and 150 mM NaCl; pH 7: 25 mM Hepes, 5 mM glucose, 1 mM MgCl, and 150 mM NaCl. Following incubation, cells were placed on ice and immediately washed twice with 0.5% fatty acid–free BSA in PBS and once with PBS. Lipids were extracted in acetonitrile:isopropanol:water of 13:6:1 (v/v/v) for 15 min and spun at 20,000*g* for 10 min at 4°C to remove cell debris. Lipid extracts were analyzed on the Agilent Triple-Quadrupole 6470 Mass Spectrometer as described below.

### d7-LPC (18:1) quantitation

Lipids were separated on an Ascentis C18 column (5 μm, 5–mum particle size, length × internal diameter, 5 cm by 4.6 mm) (Sigma-Aldrich) with an Ascentis Express guard holder and connected to a 1290 LC system. The LC system was coupled to the 6470A triple quadrupole (QQQ) mass spectrometer equipped with an LC-ESI probe. External mass calibration was performed using the standard calibration mixture every 7 days. Injection volumes of 2 to 4 μl were used for each sample, with separate injections for positive and negative ionization modes. Mobile phase A in the chromatographic method consisted of 60:40 water:acetylnitrile with 10 mM ammonium formate and 0.1% formic acid, and mobile phase B consisted of 90:10 isopropanol:acetylnitrile, with 10 mM ammonium formate and 0.1% formic acid. The chromatographic gradient was adapted from Laqtom *et al.* (*38*). Briefly, the elution was performed with a gradient of 18 min; during 0- to 1-min isocratic elution with 32% B, increase to 66% B from 1 to 6 min and increase to 75% B; from 6 to 10 min, increase to 97% B; from 10 to 14 min, solvent B was decreased to 32% and then maintained for another 4 min for column reequilibration. The flow rate was set to 0.260 ml/min. The column compressor and autosampler were held at 55° and 4°C, respectively. The mass spectrometer parameters were as follows: The spray voltage was set to 3.5 kV in a positive mode and 2.5 kV in a negative mode, and the gas temperature and the sheath gas flow were held at 250° and 300°C, respectively. Both gas flow and sheath gas flow were 12 liters/min, while the nebulizer was maintained at 25 psi. These conditions were held constant for both positive- and negative-ionization mode acquisitions.

The mass spectrometer was operated in multiple reaction method (MRM) for targeted analysis of species of interest. Standard compounds including d7-LPC (18:1), d7-LPE (18:1), d5-LPC (15:0), LPC (18:1), LPC (15:0), PI (16:0_18:1), and PC(16:0_18:0) (POPC) were purchased from Avanti Polar Lipids and optimized using a MassHunter Optimizer MRM. MassHunter Optimizer MRM is an automated method development software used to generate and optimize MRM transitions accumulating at most four products with different abundances from singly ionized species. The two most abundant transitions from either the negative or positive mode were selected to detect each species. The precursor-product ion pairs (*m*/*z*) used for MRM of the compounds were as follows: d7-LPC 18:1: 529.4 → 104.1/184.0; d7-LPE 18:1: 487.4 → 346.3/62.1; d5-LPC 15:0: 487.4 → 184.0/104.1; LPC 18:1: 522.4 → 281.3/104.1; LPC 15:0: 482.4 → 60.2/104.1; PI 16:0_18:1: 854.6 → 577.5/837.5; and POPC: 760.6 → 124.9/60.2.

High-throughput annotation and relative quantification of lipids were performed using a qualitative analysis software of MassHunter acquisition data and QQQ quantitative analysis (Quant-My-Way) software. Individual lipid species shown in the figures were validated using the Qualitative software by manually checking the peak alignment and matching the retention times and MS/MS spectra to the characteristic fragmentation compared to the standard compounds. Analyzing two transitions for the same compound and looking for similar relative response was an added validation criterion to ensure that the correct species were identified and quantified. The MRM method and retention time were used to quantify all lipid species using the quantification software, and the raw peak areas of all species were exported to Microsoft Excel for further analysis. d7-LPC raw abundances were normalized to cell number using abundance of endogenous control lipids in the same sample.

### Multiple sequence alignment

Sequences of *Homo sapiens* SPNS1 (Q9H2V7) and *D. melanogaster* Spinster (alias: bnch, Q9GQQ0) were obtained from UniProt. Clustal Omega was used to perform a multiple sequence alignment using inputted protein sequences.

### Docking with Schrödinger Glide

I-TASSER (*56*) was used to predict the structure of *H. sapiens* SPNS1 using Q9H2V7 (UniProt) as the entry sequence. The predicted *H. sapiens* SPNS1 structure was prepared in Schrödinger Glide using the Protein Preparation Wizard, which ensures structural correctness of the molecule, for example, by filling in missing residues, flipping positions of residues, adding H atoms, adding appropriate bond orders, and adding in formal charges to ensure that all atoms and residues are represented accurately in the protein. The structure of LPC 22:4 was obtained from the ZINC15 database (ZINC000040165293) and prepared using Schrödinger’s LigPrep tool to generate a three-dimensional ligand structure with energy minimization. Afterward, Schrödinger’s Receptor Grid Generation tool was used to create a grid representing the shape and properties of the receptor binding site for use in docking simulations guided by residues around the putative substrate binding site. Last, using the prepared ligand library and receptor grids, Glide Ligand Docking was performed using standard precision, which accounts for hydrophobic interactions, hydrogen bonding, and coulombic, van der Waals, and solvation terms.

### SPNS1 mutants

SPNS1 mutants SPNS1(E164K) and SPNS1(R76A) were generated by site-directed mutagenesis of the codon-optimized SPNS1 orf (see the “Plasmids” section) using the QuickChange Lightning kit (#210518, Agilent). The following primers were used for the mutagenesis (mutations to wild-type sequence in bold): SPNS1(E164K), 5′- GGGGACTGGTGGGAGTGGGA**A**AGGCAAGCTACTCCACCATCGCAC-3′ (forward) and 5’-GTGCGATGGTGGAGTAGCTTGCCTT**T**CCCACTCCCACCAGTCCCC-3′ (reverse); SPNS1(R76A), 5′-TGCTACATCAACCTGCTGAATTATATGGAT**GC**CTTTACCGTGGCAGGCGTGCTGCCAGACATCGA-3′ (forward) and 5′-TCGATGTCTGGCAGCACGCCTGCCACGGTAAAG**GC**ATCCATATAATTCAGCAGGTTGATGTAGCA-3′ (reverse).

### Mouse studies

NOD.Cg-prkdscid/J mice were purchased from The Jackson Laboratory (RRID: IMSR_JAX:001303, male, 7.5 weeks of age). Mice were maintained on a standard light-dark cycle with access to food and water ad libitum in a room with a controlled temperature (22°C) and humidity (around 50%). Mouse cages were cleaned approximately every 4 to 5 days, and supplies of water and food were checked daily. For in vivo assessment of *SPNS1*-KO tumor growth, 5 million *SPNS1*-KO or *SPNS1*-AB PaTu-8988T cells were resuspended in 100 μl of 80% Matrigel (#254534, Corning) and injected subcutaneously in the flank, with each mouse receiving bilateral injections of the same cell genotype. Each group of mice (mice with *SPNS1*-KO tumors or *SPNS1*-AB tumors) was then split into two conditions and fed choline-deficient (Research Diets Inc.) or control (Research Diets Inc.) diet, with continued access to food ad libitum. Tumor volume was calculated by caliper measurement of tumor width, length, and height (volume = width × length × height/2) every 3 days, with two gap periods, until the largest tumors reached 600 cm^3^. Mice that died or lacked tumors were omitted from the analysis. On the final day of measurements, mice were euthanized, and tumors were dissected and weighed. All the procedures involving mice were carried out in accordance with the approved guidelines by the Stanford University Administrative Panel on Laboratory Animal Care (#33464) and Institutional Animal Care and Use Committee.

### Data preparation and statistics

Displays of quantitative data were prepared in Microsoft Excel v.16.65 and GraphPad Prism v.9.0. Statistical comparisons were performed using two-tailed unpaired or paired *t* tests and ordinary one-way analysis of variance (ANOVA) with post hoc tests when relevant in Prism, unless stated otherwise in the figure legends. All displayed measurements represent samples generated independently or biological replicates unless otherwise indicated. Figure schematics were created using BioRender and were used with permission.

### Plasmids

The following sequence represents the codon-optimized SPNS1 orf used in all studies with *SPNS1*-AB condition and was derivatized to obtain SPNS1 mutants. It was cloned from Addgene pDONR221_SPNS1, a gift from RESOLUTE Consortium and Giulio Superti-Furga (plasmid no. 132273, Addgene, RRID: Addgene_132273): 5′-ATGGCCGGCTCTGACACCGCCCCTTTCCTGAGCCAGGCCGACGATCCAGACGATGGACCAGTGCCAGGAACCCCTGGACTGCCAGGCTCTACAGGCAACCCCAAGAGCGAGGAGCCAGAGGTGCCTGACCAGGAGGGACTGCAGAGGATCACAGGACTGTCCCCTGGCCGGTCTGCCCTGATCGTGGCCGTGCTGTGCTACATCAACCTGCTGAATTATATGGATCGCTTTACCGTGGCAGGCGTGCTGCCAGACATCGAGCAGTTCTTTAACATCGGCGATAGCTCCTCTGGCCTGATCCAGACAGTGTTCATCAGCTCCTACATGGTGCTGGCCCCCGTGTTCGGCTATCTGGGCGATCGCTACAATCGGAAGTATCTGATGTGCGGCGGCATCGCCTTCTGGAGCCTGGTGACCCTGGGCTCTAGCTTCATCCCAGGCGAGCACTTTTGGCTGCTGCTGCTGACAAGGGGACTGGTGGGAGTGGGAGAGGCAAGCTACTCCACCATCGCACCTACACTGATCGCCGACCTGTTTGTGGCCGATCAGAGAAGCAGGATGCTGTCCATCTTCTACTTTGCCATCCCAGTGGGAAGCGGACTGGGATATATCGCAGGATCCAAGGTGAAGGACATGGCCGGCGATTGGCACTGGGCACTGAGAGTGACCCCTGGACTGGGAGTGGTGGCCGTGCTGCTGCTGTTCCTGGTGGTGAGAGAGCCACCTAGGGGAGCAGTGGAGAGGCACTCTGACCTGCCACCCCTGAACCCAACAAGCTGGTGGGCAGATCTGCGCGCCCTGGCAAGGAATCCATCCTTCGTGCTGTCCTCTCTGGGCTTCACCGCCGTGGCCTTTGTGACAGGATCCCTGGCCCTGTGGGCACCTGCCTTTCTGCTGCGCTCTAGGGTGGTGCTGGGAGAGACCCCTCCATGCCTGCCAGGCGACTCCTGTAGCTCCTCTGATTCTCTGATCTTTGGCCTGATCACCTGCCTGACAGGCGTGCTGGGAGTGGGACTGGGAGTGGAGATCAGCCGGAGACTGAGACACTCCAACCCAAGGGCAGACCCACTGGTGTGCGCAACCGGACTGCTGGGCTCCGCCCCCTTCCTGTTTCTGTCTCTGGCATGTGCAAGGGGAAGCATCGTGGCAACCTACATCTTCATCTTTATCGGCGAGACACTGCTGAGCATGAATTGGGCCATCGTGGCCGATATCCTGCTGTATGTGGTCATCCCAACCAGGCGCTCCACAGCCGAGGCCTTCCAGATCGTGCTGTCTCACCTGCTGGGCGACGCAGGAAGCCCATATCTGATCGGCCTGATCTCCGATCGCCTGCGGAGAAATTGGCCCCCTTCTTTCCTGAGCGAGTTTCGGGCCCTGCAGTTCAGCCTGATGCTGTGCGCCTTTGTGGGCGCCCTGGGAGGAGCAGCCTTCCTGGGCACCGCCATCTTTATCGAGGCCGACAGGCGCCGGGCACAGCTGCACGTGCAGGGACTGCTGCACGAGGCAGGCTCCACCGACGATAGAATCGTGGTGCCCCAGAGAGGCAGGTCCACAAGGGTGCCTGTGGCCTCTGTGCTGATC-3′.

In all plasmids, the N terminus includes 3xFlag with linker or 3xFlag-GFP with linker and the C terminus retains a short 19 amino acid linker from the vector backbone (sequence: VSGRYVNSAPPPLSLPPP).

Plasmids used in this manuscript are as follows: PX458-SPNS1-sg1 (Addgene ID: 198566), PX458-SPNS1-sg2 (Addgene ID: 198567), pMXs-IRES-Blast3xFlag-SPNS1, pMXs-IRES-Blast-3xFlag-SPNS1(E164K), pMXs-IRES-Blast-3xFlag-SPNS1(R76A), pMXs-IRES-Blast-3xFlag-GFP-SPNS1, pMXs-IRES-Blast-3xFlag-GFP-SPNS1(E164K), pMXs-IRES-Blast-3xFlag-GFP-SPNS1(R76A), and pLJC5-TMEM192-3xHA (Addgene ID: 102930) (*4*).

## Notes

View/request a protocol for this paper from *Bio-protocol*.
