# Supplementary material for: An SPNS1-dependent lysosomal lipid transport pathway that enables cell survival under choline limitation
Source: Sci Adv. 2023 Apr 19;9(16):eadf8966. doi: 10.1126/sciadv.adf8966 (PMC10115416; doi:10.1126/sciadv.adf8966)
Supplement: Supplementary file 1 — Figs. S1 to S5 Legends for tables S1 to S3 [file sciadv.adf8966_sm.pdf]

Science Advances
AAAS

Supplementary Materials for

An SPNS1-dependent lysosomal lipid transport pathway that enables cell survival under choline limitation

Samantha G. Scharenberg *et al.*

Corresponding author: Monther Abu-Remaileh, monther@stanford.edu

Sci. Adv. **9**, eadf8966 (2023)
DOI: 10.1126/sciadv.adf8966

The PDF file includes:

Figs. S1 to S5
Legends for tables S1 to S3

Other Supplementary Material for this manuscript includes the following:

Tables S1 to S3

Figure S1

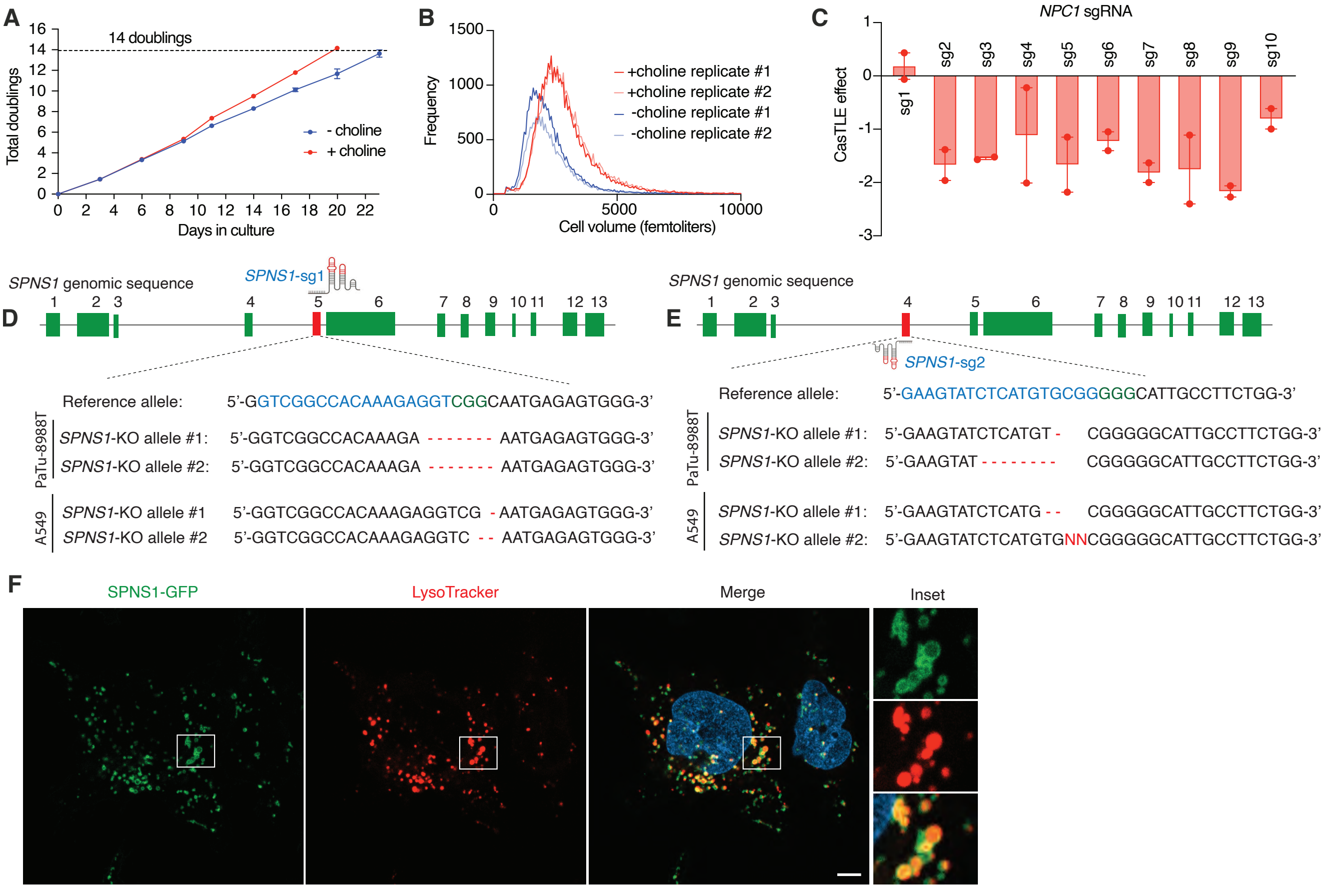

Supplementary Figure 1: Endolysosomal CRISPR-Cas9 screen implicates SPNS1 in lysosomal choline recycling
A) Cell doublings vs. time for PaTu-8988T pancreatic cancer cells grown in choline-depleted medium (-choline, blue) or medium supplemented with 100 µM choline (+choline, red). Data show mean +/- SEM ($n = 2$). **B)** Distribution of cell volumes after culturing for the indicated days in choline-depleted medium (-choline, blue) or medium supplemented with 100 µM choline (+choline, red). Data in **(A)** and **(B)** are derived from the actual screen replicates. **C)** Individual CasTLE effects in -choline vs. +choline for all *NPC1*-targeting sgRNAs used in the endolysosomal library. Data show mean +/- SEM ($n = 2$). **D)** Schematic illustrating the genomic target of *SPNS1* sgRNA-1 in exon 5 of the human *SPNS1* locus and sequences of the mutated alleles of PaTu-8988T-KO1 and A549-KO1 clones. **E)** Schematic illustrating the genomic target of *SPNS1* sgRNA-2 in exon 4 of the human *SPNS1* locus and sequences of the mutated alleles of PaTu-8988T-KO2 and A549-KO2 clones. **F)** Fluorescence microscopy images of PaTu-8988T cells expressing SPNS1-GFP. SPNS1-GFP is imaged in the green channel (left). Lysosomes are stained with LysoTracker DND-99 and imaged in the red channel (middle).The merged image (right) demonstrates co-localization of SPNS1-GFP with lysosomes. White scale bar represents 5 µm.

Figure S2

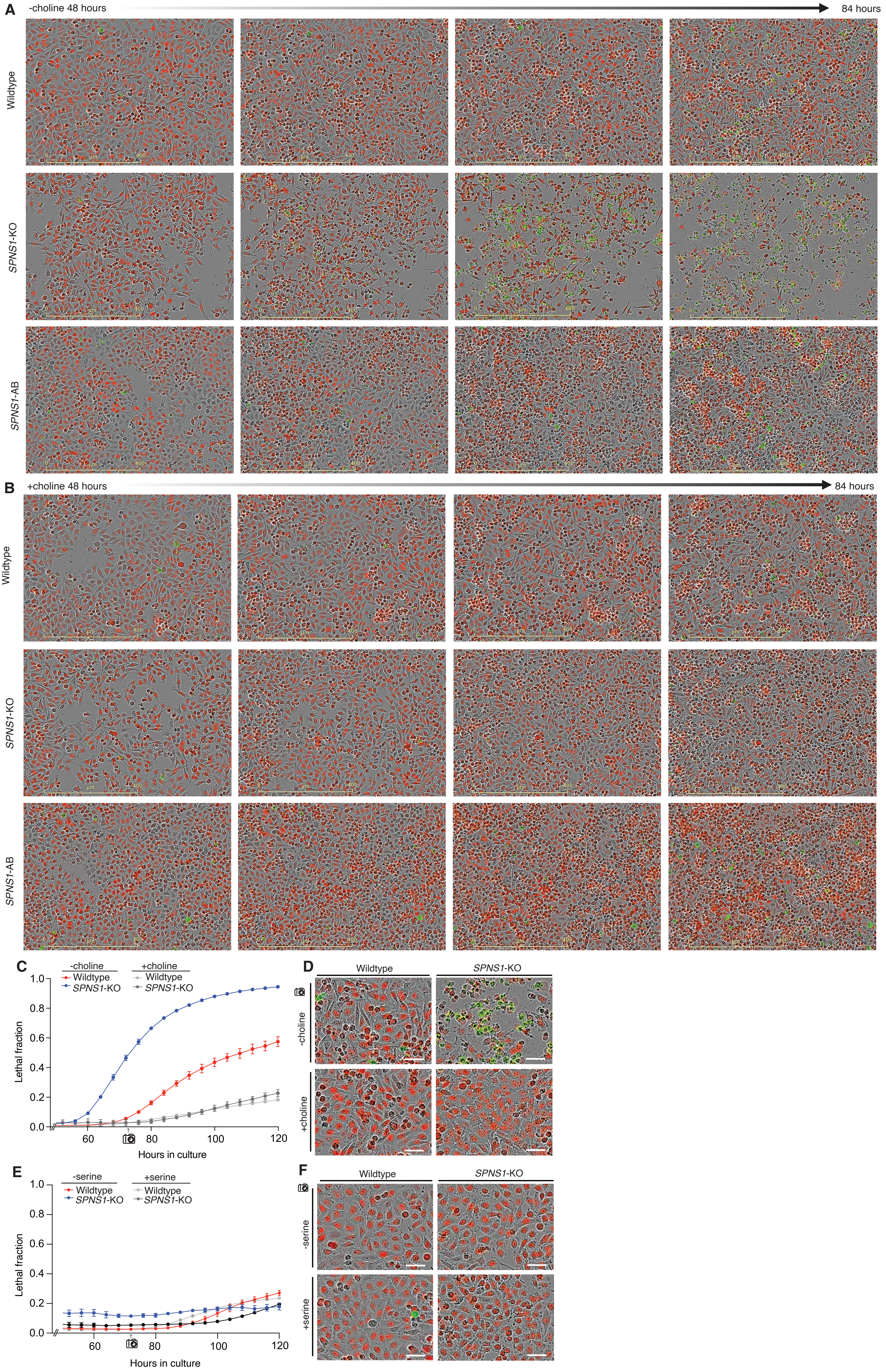
A
-choline 48 hours
84 hours
Wildtype
SPNS1-KO
SPNS1-AB
B
+choline 48 hours
84 hours
Wildtype
SPNS1-KO
SPNS1-AB
C
-choline
Wildtype
SPNS1-KO
+choline
Wildtype
SPNS1-KO
Lethal fraction
Hours in culture
D
Wildtype
SPNS1-KO
-choline
+choline
E
-serine
Wildtype
SPNS1-KO
+serine
Wildtype
SPNS1-KO
Lethal fraction
Hours in culture
F
Wildtype
SPNS1-KO
-serine
+serine

Supplementary Figure 2: *SPNS1* deficiency promotes cell death under choline-limiting conditions

A&B) Representative fields from the Incucyte lethal fraction analysis depicting a time-course of wildtype, *SPNS1*-KO and *SPNS1*-AB cells during -choline culture **(A)** and +choline culture **(B)** from 48 to 84 hours. Green marks dead cells; red marks nuclei of live cells. From same experiment in **Fig. 2C&D. C)** Lethal fraction of PaTu-8988T wildtype and another *SPNS1*-KO cell clone grown in choline-depleted medium (-choline) or medium supplemented with 100 μM choline chloride (+choline). Data show mean +/- SEM (*n* = 3). **D)** Representative fields from **(C)** at the 72-hour time-point. Green marks dead cells; red marks nuclei of live cells. White scale bar represents 50 μm. **E)** Lethal fraction of wildtype and *SPNS1*-KO cells (same KO clone as in **(C)**) grown in serine-depleted medium (-serine) or medium supplemented with 27 mg/L serine (+serine). Data show mean +/- SEM (*n* = 3). **F)** Representative Incucyte fields from **(E)** at the 72-hour time-point. Green marks dead cells; red marks nuclei of live cells. White scale bar represents 50 μm.

Figure S3

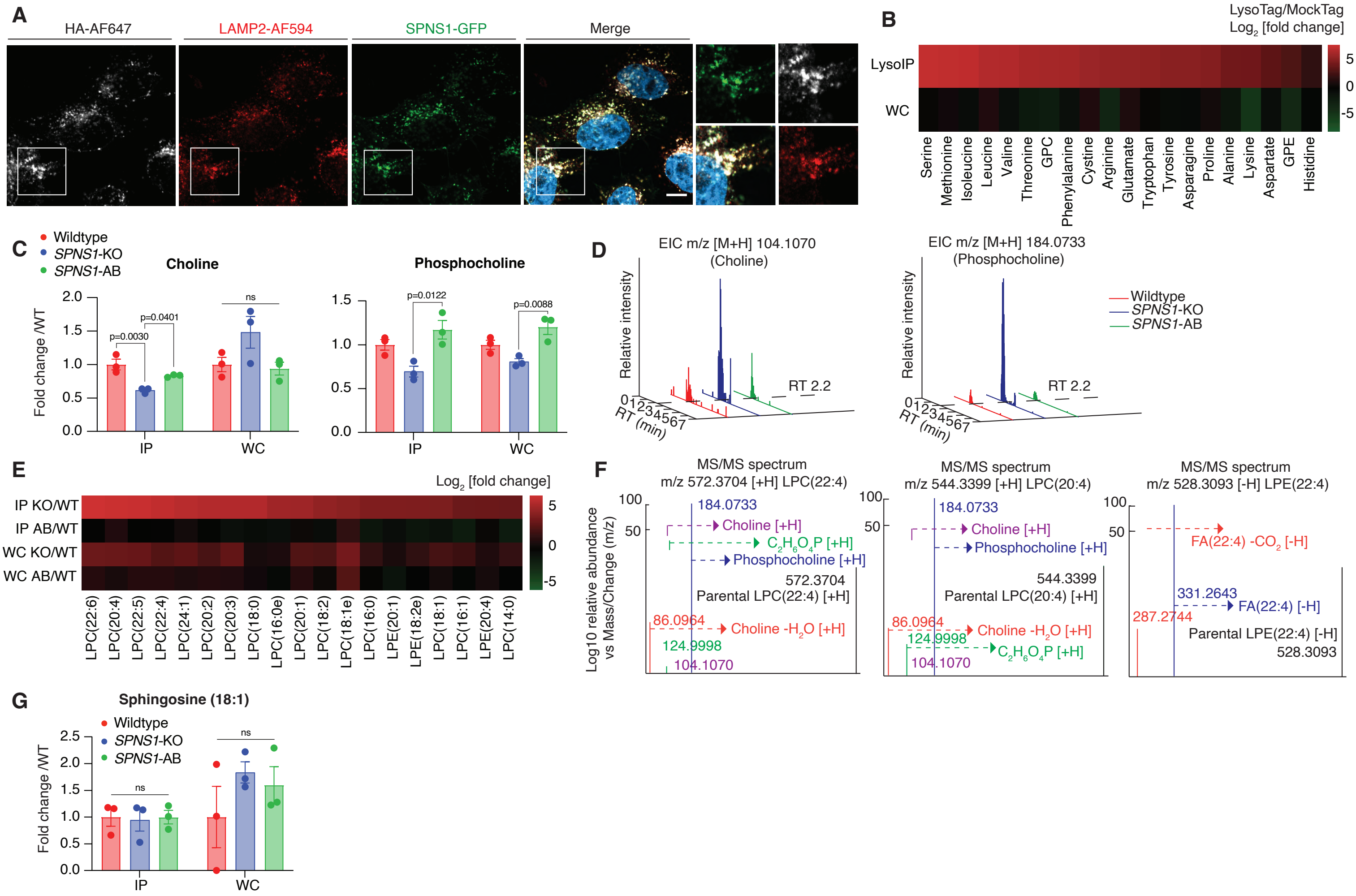

Supplementary Figure 3: SPNS1-deficient lysosomes accumulate LPCs and LPEs

A) Representative fluorescence microscopy images of PaTu-8988T *SPNS1*-AB cells infected with LysoTag (TMEM192-3xHA). The image depicts HA (LysoTag) staining with AF-647 (white), lysosomal marker LAMP2 staining with AF-598 (red), SPNS1-GFP (green), and a merged image demonstrating co-localization of LysoTag, LAMP2 and SPNS1-GFP. Nuclei are stained with Hoechst (blue). White scale bar represents 5 µm. **B)** Heat map depicting relative abundances of known lysosomal-enriched metabolites in whole cell (WC) and lysosomes (LysoIP) of wildtype PaTu-8988T cells with LysoTag (TMEM192-3xHA; $n = 3$) versus MockTag (TMEM192-3xflag; $n = 1$). **C)** Quantitation of lysosomal (IP) and whole-cell (WC) abundance of choline and phosphocholine (normalized to a pool of endogenous amino acids: phenylalanine, methionine and tyrosine in positive ion mode) in wildtype, *SPNS1*-KO1 and *SPNS1*-AB1 cells. Data show fold change of mean of metabolite abundance versus wildtype +/- SEM ($n = 3$). Statistical test: one-way ANOVA with Tukey's HSD post-hoc. **D)** Extracted ion chromatograms (EICs) for choline and phosphocholine produced through in-source fragmentation of lysophosphatidylcholines in wildtype, *SPNS1*-KO and *SPNS1*-AB cells. **E)** Heatmap depicting fold changes in the abundance of LPC and LPE species from hydrophilic interaction liquid chromatography (HILIC, for polar metabolites) measurement in the indicated cells. Within each column (lipid species) for each fraction (IP and WC), fold changes were calculated by normalizing to the levels in the wildtype (WT) ($n = 3$). **F)** Annotated tandem mass spectra validating the detection of selected LPCs and LPE. Relative abundance is shown in logarithm scale for better visualization of characteristic fragments with low intensity. **G)** Targeted quantitation of sphingosine (18:1) in wildtype, *SPNS1*-KO and *SPNS1*-AB cells demonstrating no accumulation in *SPNS1*-KO lysosomes (IP) and cells (WC). Data show mean +/- SEM ($n = 3$). Statistical test: one-way ANOVA with Tukey's HSD post-hoc.

Figure S4

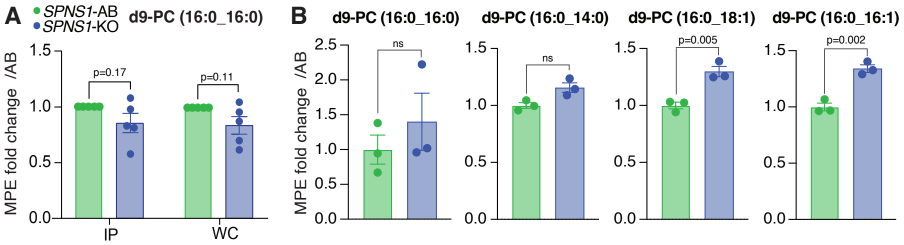

Supplementary Figure 4: SPNS1 is required for lysosomal efflux of LPC

A) Quantitation of tracer d9-PC (16:0_16:0) in lysosomes (IP) and whole cells (WC) of *SPNS1*-KO and *SPNS1*-AB cells expressed as paired-sample fold-change. Data show mean +/- SEM (n = 5). Statistical test: two-tailed paired t-test. Data belong to experiments presented in **Fig. 4C-D**. **B)** Quantitation of molar percent enrichment (MPE) for selected phosphatidylcholines at the whole-cell level in *SPNS1*-KO and *SPNS1*-AB cells. Cells were treated with d9-choline for 3 hours in media depleted of unlabeled choline. Data shown as mean +/- SEM (n = 3). Statistical test: two-tailed unpaired t-test.

Figure S5

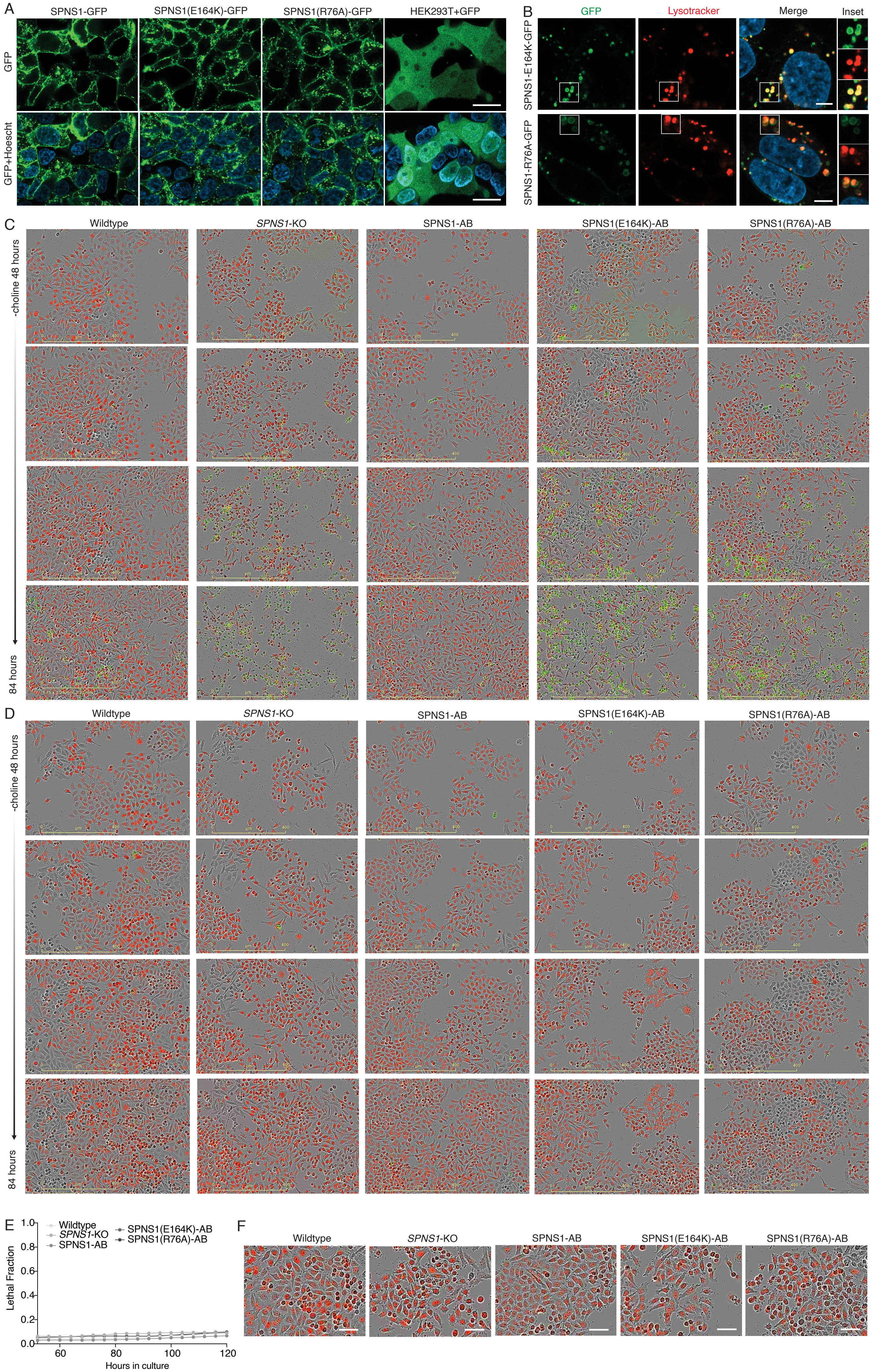
A
SPNS1-GFP
SPNS1(E164K)-GFP
SPNS1(R76A)-GFP
HEK293T+GFP
GFP
GFP+Hoescht
B
GFP
Lysotracker
Merge
Inset
SPNS1-E164K-GFP
SPNS1-R76A-GFP
C
Wildtype
SPNS1-KO
SPNS1-AB
SPNS1(E164K)-AB
SPNS1(R76A)-AB
-choline 48 hours
84 hours
D
Wildtype
SPNS1-KO
SPNS1-AB
SPNS1(E164K)-AB
SPNS1(R76A)-AB
-choline 48 hours
84 hours
E
Wildtype
SPNS1-KO
SPNS1-AB
SPNS1(E164K)-AB
SPNS1(R76A)-AB
Lethal Fraction
1.0
0.8
0.6
0.4
0.2
0.0
60
80
100
120
Hours in culture
F
Wildtype
SPNS1-KO
SPNS1-AB
SPNS1(E164K)-AB
SPNS1(R76A)-AB

Supplementary Figure 5: LPC efflux from lysosomes mediates cell survival under choline limitation

A) Representative fluorescence microscopy images of HEK293T cells over-expressing SPNS1-GFP, SPNS1(E164K)-GFP, SPNS1(R76A)-GFP and soluble GFP. Nuclei are stained with Hoechst (blue). White scale bar represents 20 µm. **B)** Representative fluorescence microscopy images of PaTu-8988T cells demonstrating co-localization of SPNS1(E164K)-GFP and SPNS1(R76A)-GFP (green) with Lysotracker (red). Nuclei are stained with Hoechst (blue). White scale bar represents 5 µm. **C&D)** Representative fields from the Incucyte lethal fraction analysis depicting a time-course of SPNS1 (E164K)-AB, SPNS1(R76A)-AB, and SPNS1-AB cells during -choline culture **(C)** and +choline culture **(D)** from 48 to 84 hours. Green marks dead cells; red marks nuclei of live cells. From same experiment in **Fig. 5D&E**. **E)** Lethal fraction of Wildtype, *SPNS1*-KO, *SPNS1*-AB, and *SPNS1*-AB mutants grown in control (+choline) medium. Data show mean +/- SEM (*n* = 3). **F)** Representative Incucyte fields from **(E)** at the 72-hour time-point. Green marks dead cells; red marks nuclei of live cells. White scale bar represents 50 µm.

Supplementary tables

Supplementary table 1: Endolysosomal library sgRNA sequences

Supplementary table 2: Ranking of all gene CasTLE effects and scores computed between -choline and +choline conditions in the endolysosomal screen

Supplementary table 3: Untargeted lipidomics of the lysosomes and whole cells derived from *SPNS1*-KO and wildtype PaTu-8988T cells
